# A Primeval Mechanism of Tolerance to Desiccation Based on Glycolic Acid Saves Neurons in Mammals from Ischemia by Reducing Intracellular Calcium‐Mediated Excitotoxicity

**DOI:** 10.1002/advs.202103265

**Published:** 2021-12-14

**Authors:** Alexandra Chovsepian, Daniel Berchtold, Katarzyna Winek, Uta Mamrak, Inés Ramírez Álvarez, Yanina Dening, Dominika Golubczyk, Luis Weitbrecht, Claudia Dames, Marine Aillery, Celia Fernandez‐Sanz, Zdzislaw Gajewski, Marianne Dieterich, Miroslaw Janowski, Peter Falkai, Piotr Walczak, Nikolaus Plesnila, Andreas Meisel, Francisco Pan‐Montojo

**Affiliations:** ^1^ Department of Psychiatry and Psychotherapy Ludwig‐Maximilian University Hospital Nussbaumstrasse. 7 80336 Munich Germany; ^2^ Department of Neurology NeuroCure Clinical Research Center Center for Stroke Research Charité University Medicine Charitéplatz 1 10117 Berlin Germany; ^3^ Laboratory of Experimental Stroke Research Institute for Stroke and Dementia Research (ISD) University of Munich Medical Center Feodor‐Lynen‐Strasse 17 81377 Munich Germany; ^4^ Department of Neurology Ludwig‐Maximilian University Hospital Marchioninstrasse. 15 81377 Munich Germany; ^5^ Munich Cluster for Systems Neurology (SyNergy) Ludwig‐Maximilian University Munich 81377 Munich Germany; ^6^ Ti‐com LLC Trylinskiego 2 Olsztyn 10‐683 Poland; ^7^ Center for Translational Medicine Warsaw University of Life Sciences Warsaw 02‐787 Poland; ^8^ Program in Image Guided Neurointerventions Department of Diagnostic Radiology and Nuclear Medicine University of Maryland Baltimore MD 21201 USA; ^9^ Present address: Present address: Edmond and Lily Safra Center for Brain Sciences Hebrew University of Jerusalem Jerusalem 9190401 Israel; ^10^ Present address: Present address: Seppic Île‐de‐France La Garenne‐Colombes 92250 France; ^11^ Present address: Present address: Center for Translational Medicine Department of Medicine Thomas Jefferson University Philadelphia PA 19107 USA

**Keywords:** glutamate‐dependent excitotoxicity, ischemia–reperfusion damage, neuroprotection, Stroke

## Abstract

Stroke is the second leading cause of death and disability worldwide. Current treatments, such as pharmacological thrombolysis or mechanical thrombectomy, reopen occluded arteries but do not protect against ischemia‐induced damage that occurs before reperfusion or neuronal damage induced by ischemia/reperfusion. It has been shown that disrupting the conversion of glyoxal to glycolic acid (GA) results in a decreased tolerance to anhydrobiosis in *Caenorhabditis elegans* dauer larva and that GA itself can rescue this phenotype. During the process of desiccation/rehydration, a metabolic stop/start similar to the one observed during ischemia/reperfusion occurs. In this study, the protective effect of GA is tested in different ischemia models, i.e., in commonly used stroke models in mice and swine. The results show that GA, given during reperfusion, strongly protects against ischemic damage and improves functional outcome. Evidence that GA exerts its effect by counteracting the glutamate‐dependent increase in intracellular calcium during excitotoxicity is provided. These results suggest that GA treatment has the potential to reduce mortality and disability in stroke patients.

## Graphical abstract

In this paper, it is shown how the survival strategy of the worm *Caenorhabditis elegans* against desiccation, namely, producing glycolic acid at high concentrations, can be used to protect against stroke in mammals. Glycolic acid mitigates the deleterious effects of ischemia/reperfusion by decreasing the glutamate‐dependent abnormal calcium influx to the cells, leading to reduced lesion sizes in mice and swine.

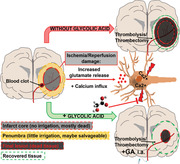

## 1 Introduction

Stroke is the second leading cause of death and disability worldwide and is responsible for ≈5.5 million deaths and 116.4 million disability‐adjusted life years globally.^[^ ^1^ ^]^ Ischemic stroke comprises 87% of all stroke cases^[^ ^2^ ^]^ and is caused by a thrombotic or embolic vessel occlusion, which results in focal cerebral ischemia. It takes only minutes for the affected area of the brain to become irreversibly damaged, often resulting in long‐term neurological deficits (such deficits occur in 50% to 60% of stroke patients^[^ ^3^ ^]^). Blood flow to the tissue surrounding the necrotic infarct core is reduced, but this tissue, which is referred to as the penumbra, is salvageable because of collateral perfusion.^[^ ^4^ ^]^ The penumbra is thus the main target of current treatment strategies.^[^ ^5^ ^]^

Several treatments have been tested after stroke, including *N*‐methyl‐d‐aspartate (NMDA) and NMDA antagonists,^[^ ^6^ ^]^ free radical scavengers,^[^ ^7^, ^8^ ^]^ and immunomodulators (tumor necrosis factor alpha,^[^ ^9^ ^]^ interleukin‐10^[^ ^10^ ^]^). However, no treatment has been successful in the preclinical or clinical phase apart from the free radical scavenger edaravone, which received clinical approval in Japan.^[^ ^11^ ^]^ Hence, identifying novel neuroprotective compounds is essential for improving treatment options in ischemic stroke.

The inherent ability of *Caenorhabditis elegans* to tolerate extreme desiccation in the dauer larva stage, a special diapause stage that occurs in unfavorable environmental conditions, may have interesting implications in stroke. The strategies that enable the *C. elegans* dauer larva to survive desiccation have been extensively studied.^[^ ^12^, ^13^, ^14^, ^15^ ^]^ Desiccation and rehydration represent a form of metabolic stress (metabolic stop/start)^[^ ^16^ ^]^ and mirror the metabolic halt and reactivation that occur during ischemia–reperfusion damage due to restriction of blood flow, oxygen, and nutrients,^[^ ^17^, ^18^ ^]^ followed by an abrupt reinitiation of metabolic activity during rehydration/reperfusion. Research has been shown that *djr‐1.1*, *djr‐1.2*, and *glod‐4* are strongly upregulated in dauer larva during preparation for desiccation and that these genes are crucial for desiccation tolerance.^[^ ^14^, ^15^, ^19^ ^]^ *djr‐1.1* and *djr‐1.2* are orthologs of the Parkinson's disease‐associated glyoxalase DJ‐1 (locus PARK 7), which is known to convert the reactive aldehydes glyoxal and methylglyoxal to glycolic acid (GA) and d‐lactic acid (DL), respectively.^[^ ^20^, ^21^ ^]^ A previous study^[^ ^19^ ^]^ showed that mutant worms without any glyoxalase activity (i.e., that lacked *djr* and *glod‐4* genes) were less likely to survive desiccation. Moreover, mitochondria of *djr‐1.1*; *djr‐1.2* double mutant larvae and DJ‐1‐knockdown HeLa cells showed defects in their network structure and membrane potential. These phenotypes were rescued by GA and DL, demonstrating that the aforementioned deficits are not just a result of the accumulation of toxic aldehydes but are also caused by the absence of the DJ‐1 glyoxalase activity products GA and DL themselves.^[^ ^19^ ^]^

On the basis of these findings and the similarity between desiccation–rehydration and ischemia–reperfusion,^[^ ^16^, ^17^, ^18^ ^]^ we hypothesized that GA and DL might protect against damage caused by ischemic stroke. Therefore, we tested these substances in vitro by oxygen–glucose deprivation (OGD) and in vivo by global cerebral ischemia (GCI) and middle cerebral artery occlusion (MCAO) in mice and in an endovascular stroke model in swine. Overall, our results showed that GA treatment exerts powerful protection against ischemia and improves functional outcome.

## 2 Results

### 2.1 GA Protects from Ischemia‐Induced Neuronal Death and Reduces Glutamate‐Dependent [Ca^2+^] Influx in Cortical Neurons In Vitro

**Figure 1:**
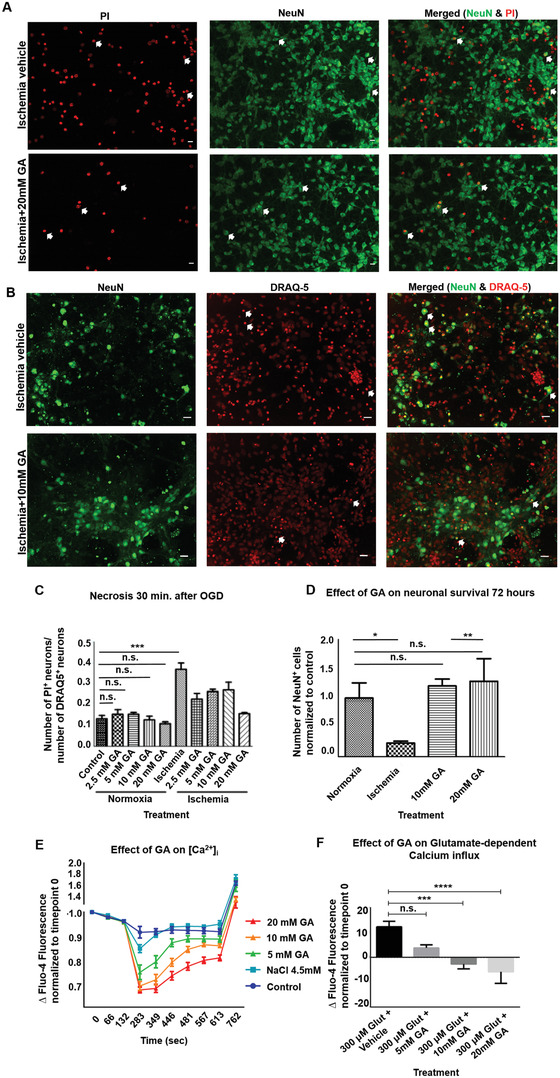
Glycolic acid reduces necrosis and apoptosis in the oxygen–glucose deprivation model by reducing intracellular calcium. A) Representative fluorescence microscopy images of neurons stained with propidium iodide (PI, left panel, red) during reperfusion to evaluate necrosis and imaged 30 min after reperfusion. The middle panel shows the same neuronal population stained for neuronal nuclei (NeuN, green) to label all neuronal bodies, and the right panel shows both stainings together. White arrows indicate examples of necrotic (PI^+^, NeuN^+^) neurons. Upper panel: Neurons that underwent ischemia and were treated with vehicle containing reperfusion medium (ischemia + vehicle). Lower panel: Neurons that underwent ischemia and were treated with reperfusion medium containing 20 × 10^−3^ m glycolic acid [GA; (ischemia+20 × 10^−3^ m GA)]. B) Representative fluorescence microscopy images of neurons stained for NeuN (left panel, green) and DRAQ5 nuclear labeling (middle panel, red) 72 h after oxygen–glucose deprivation (OGD) and reperfusion. The right panel shows the merge of the two stainings. White arrows show examples of pyknotic nuclei that are identified by overaccumulation of DRAQ5 and their small size. Upper panel: ischemia + vehicle. Lower panel: ischemia + 10 × 10^−3^ m GA. The figure shows that neuron morphology was severely damaged in the vehicle group, which had affected or absent neurites, but was more conserved in the GA‐treated group. Three biological replicates (*n* = 3) were used, consisting of 5 technical replicates each (mean of 5 wells per condition per plate). Scale bars: 20 µm. C) GA at all tested concentrations significantly reduced the levels of necrosis at 30 min after OGD, as measured by the ratio of PI^+^ (necrotic) neurons to NeuN^+^ (non‐necrotic) neurons. The strongest effect was achieved by 20 × 10^−3^ m GA (one‐way analysis of variance [ANOVA] followed by Tukey's multiple comparison's test, *p* < 0.0001). D) GA treatment during reperfusion protected cortical neurons against 60 min ischemia‐induced cell death, up to 72 h after the insult. Numbers are normalized to normoxia (control); an unpaired *t*‐test was performed. Data are presented as mean ± SEM; n.s: nonsignificant, *p < 0.05, **p < 0.01, ***p < 0.001, and ****p < 0.0001. E) The graph shows the effect of 5 × 10^−3^, 10 × 10^−3^, and 20 × 10^−3^ m GA on intracellular calcium levels (here, expressed as ΔFluo‐4‐fluorescence normalized to timepoint 0) before and after addition of 2 × 10^−6^ m ionomycin in mouse cortical neurons loaded with 5 × 10^−6^ m Fluo‐4‐AM. Two‐way ANOVA followed by Sidak's multiple comparisons test: control versus 5 × 10^−3^ m GA, *p* < 0.001; control versus 10 × 10^−3^ m GA, *p* < 0.0001; control versus 20 × 10^−3^ m GA, *p* < 0.0001; control versus 4.5 × 10^−3^ m NaCl, n.s.; 4.5 × 10^−3^ m NaCl versus 5 × 10^−3^ m GA, *p* < 0.0001; 4.5 × 10^−3^ m NaCl versus 10 × 10^−3^ m GA, *p* < 0.0001; 4.5 × 10^−3^ m NaCl versus 20 × 10^−3^ m GA, *p* < 0.0001; 5 × 10^−3^ m GA versus 10 × 10^−3^ m GA, *p* < 0.0001; 5 × 10^−3^ m GA versus 20 × 10^−3^ m GA, *p* < 0.0001; and 10 × 10^−3^ m GA versus 20 × 10^−3^ m GA, *p* = 0.0018. F) The graph shows the effect of GA on glutamate‐dependent calcium influx, measured as ΔFluo‐4‐fluorescence normalized to the timepoint pretreatment. One‐way ANOVA followed by Tukey's multiple comparisons test: vehicle versus 5 × 10^−3^ m GA, n.s.; vehicle versus 10 × 10^−3^ m GA, *p* < 0.001; vehicle versus 20 × 10^−3^ m GA, *p* < 0.0001; 5 × 10^−3^ m GA versus 10 × 10^−3^ m GA, n.s.; 5 × 10^−3^ m GA versus 20 × 10^−3^ m GA, *p* < 0.05; and 10 × 10^−3^ m GA versus 20 × 10^−3^ m GA, n.s. GA, glycolic acid; Glut, glutamate; PI, propidium iodide; NeuN, total neuronal nuclei.

The current standard of care for treating acute stroke is to restore blood flow to the ischemic region, known as reperfusion. However, this procedure can also cause damage, an effect known as “ischemia–reperfusion injury.”^[^ ^22^ ^]^ Oxygen–glucose deprivation is a well‐established in vitro model mimicking ischemia/reperfusion (IR) injury, leading to apoptotic and excitotoxicity‐induced necrotic and apoptotic cell death.^[^ ^23^ ^]^ Therefore, we used this model to determine the neuroprotective properties of GA during ischemia–reperfusion damage by testing the effects of GA in different concentrations added to the culture media immediately after mimicking 1 h of ischemia. Briefly, on day in vitro (DIV) 7, normoxic medium was replaced by either normoxic medium (normal medium) or ischemic medium (phosphate‐buffered saline (PBS), pH 6.4, bubble in N_2_) and neurons were placed for 1 h in either the incubator again (normoxia) or an anoxic environment consisting of an N_2_‐filled gas chamber; both the incubator and chamber were held at 37 °C. To mimic reperfusion, the anoxic buffer was washed out and replaced by normal medium supplemented with water as vehicle or GA at final concentrations of 2.5 × 10^−3^, 5 × 10^−3^, 10 × 10^−3^ , or 20 × 10^−3^ m. For the normoxic group, neurons were kept at 5% CO_2_ for 1 h, and then the medium was replaced again with normal medium. To differentiate between necrosis and apoptosis, we used two different approaches. Necrosis just after the ischemic insult was assessed as the ratio of necrotic propidium iodide positive (PI^+^) cells to the total number of neuronal nuclei positive (NeuN)^+^ cells 30 min after OGD compared to normoxic neurons treated with the same concentrations of GA. We observed a significant increase in the number of PI^+^ nuclei in the neuronal cultures that underwent OGD compared with the number in the normoxic cultures (OGD, 0.368 ± 0.059; normoxic, 0.132 ± 0.030; *p* < 0.001; one‐way analysis of variance (ANOVA) followed by Tukey's multiple comparisons test; **Figure** **1**A,C). Upon OGD, treatment with GA during reperfusion decreased necrotic nuclei/total nuclei ratio compared to vehicle treatment; the highest effect was observed with the 20 × 10^−3^ m concentration (OGD + 20 × 10^−3^ m GA: 0.156 ± 0.008, *p* < 0.001). In the normoxia group, the various concentrations of GA had no effect compared with the normoxia vehicle, even at the highest concentrations (normoxia + 20 × 10^−3^ m GA: 0.108 ± 0.021, *p* > 0.05). We then evaluated the number of NeuN^+^ neurons at 72 h after OGD to evaluate the total cell death (i.e., combined cell death from necrosis that occurs at early stages after OGD and apoptosis that takes place at later stages). As shown in Figure 1, OGD resulted in the loss of 76% of NeuN^+^ cells when compared with normoxia (OGD, 255.3 ± 35.32; normoxia, 1092 NeuN^+^ cells in 10 fields per well ± 284.0; *p* < 0.05; unpaired *t*‐test; Figure 1B,D). Treatment with 10 × 10^−3^ and 20 × 10^−3^ m GA immediately after OGD exerted a clear protection, increased the number of surviving neurons to levels comparable to those found with normoxia (OGD + 10 × 10^−3^ m GA, 1230 ± 154.8, *p* = 0.693; OGD + 20 × 10^−3^ m GA, 1404 ± 422.5, *p* = 0.573). We found no significant difference in neuronal survival between treatment with 10 × 10^−3^ and 20 × 10^−3^ m GA (*p* = 0.718). These results show that treatment with 10 × 10^−3^ or 20 × 10^−3^ m GA during reperfusion rescued neurons from ischemia‐induced cell death after OGD. Excitotoxicity is a major mechanism underlying neuronal death in stroke and is caused by the abnormally high calcium influx into cells via NMDA receptors (also known as glutamate‐dependent excitotoxicity).^[^ ^24^ ^]^ We previously described that GA reduces intracellular calcium in HeLa cells.^[^ ^25^ ^]^ Therefore, we decided to investigate whether it also reduces intracellular calcium in cortical neurons in vitro, even in the presence of glutamate. To that end, we tested the effect of GA on primary cortical neuronal cultures from mouse embryos loaded with Fluo‐4‐AM, an intracellular calcium indicator. With the help of a plate reader, intracellular calcium concentrations inside mouse cortical neurons ([Ca^2+^]i) were recorded at different time points before and after the addition of GA and ionomycin (Figure 1E). We found that increasing concentrations of GA (5 × 10^−3^, 10 × 10^−3^, and 20 × 10^−3^ m) significantly reduced the intracellular calcium compared with vehicle (PBS) and the osmolarity control 4.5 × 10^−3^ m NaCl (two‐way ANOVA, all *p* < 0.0001), with 20 × 10^−3^ m GA showing the strongest effect. Addition of ionomycin just before the measurement at 762 s strongly increased the influx of calcium into the cells, but 20 × 10^−3^ and 10 × 10^−3^ m of GA were able to keep the calcium levels lower than control and 4.5 × 10^−3^ m NaCl‐treated neurons (one‐way ANOVA_762 s_, *p* = 0.0011). We then tested whether GA reduced the calcium influx even in the presence of a high concentration of glutamate (300 × 10^−6^ m), which mimicked excitotoxicity. Indeed, our results show that the higher concentrations of GA (10 × 10^−3^ and 20 × 10^−3^ m) significantly decreased [Ca^2+^]i in cortical neurons in the presence of glutamate (one‐way ANOVA, *p* < 0.0001) (Figure 1F). Taken together, these data indicate that GA can mitigate the deleterious intracellular calcium increases that are associated with ischemia/reperfusion damage.

### 2.2 Unilateral Intra‐Arterial (i.a.) Administration of GA during Reperfusion Protects against Neuronal Death in the Ipsilateral CA1 Region of the Hippocampus during GCI in Mice

On the basis of these results, we decided to evaluate the effect of GA in a well‐established in vivo model of GCI. GCI occurs in case of a pronounced drop in the oxygen or blood supply to the brain (e.g., due to myocardial infarction, heart arrhythmias, sudden and prolonged decrease in blood pressure, insufficient oxygen or blood supply in protracted or complicated labor, and strangulation by the umbilical cord), which leads to anoxic/hypoxic brain damage. In adult patients, 5 min of anoxia are sufficient to cause significant neuronal metabolic impairment, leading to permanent brain damage and, in some cases, coma or even death.^[^ ^26^ ^]^

**Figure 2:**
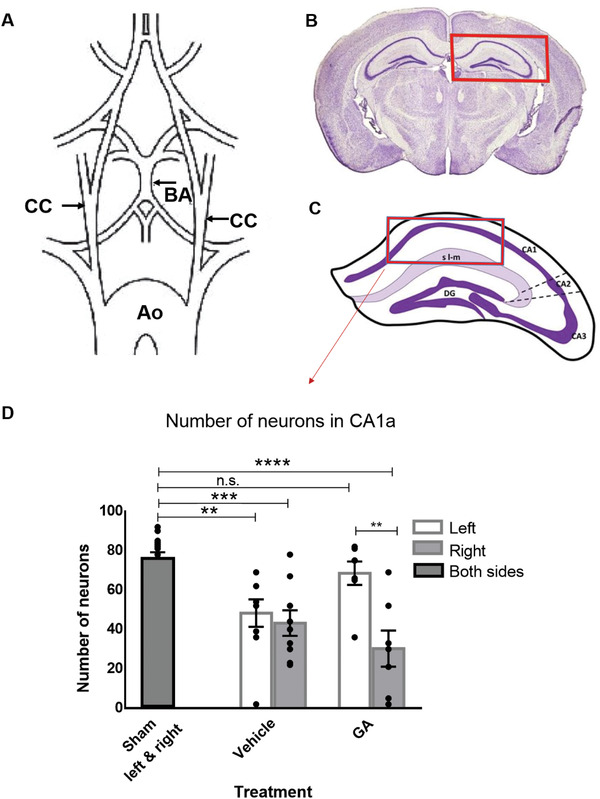
Glycolic acid increases neuronal survival in the hippocampus of a global cerebral ischemia mouse model. A) Schematic illustration of the arterial brain supply indicating (arrows) the positions of transient clipping (for 7.5 min) to induce of global cerebral ischemia (GCI). B) GCI model affects the hippocampus, shown in the red rectangle. C) Neurons were quantified inside the area indicated by the red rectangle (cornu ammonis 1 [CA1] area). D) Number of neurons quantified after administration of 50 µL glycolic acid (GA) or phosphate‐buffered serum (PBS) immediately after ischemia at the moment of reperfusion through a catheter placed in the left carotid artery. The number of surviving neurons in the left CA1 was significantly lower in PBS‐treated animals than in sham animals, but no significant difference was observed for the GA‐treated group. In contrast, the right CA1, i.e., contralateral to the injection site, showed significant neuronal death in both PBS‐ and GA‐treated animals, underscoring that the neuroprotective effect of GA is local. One‐way analysis of variance followed by Dunnett's multiple comparisons test, ^**^ *p* < 0.01, ^***^ *p* < 0.001, and ^****^ *p* < 0.0001. Data shown as mean ± SEM; *n*_sham_ = 16, *n*_veh_ = 9, and *n*_GA_ = 7. Ao, aorta; BA, basilar artery; CA1a, cornu ammonis 1a; CC, common carotid artery.

For these experiments, we used the previously described mouse model of GCI^[^ ^27^, ^28^ ^]^ to test the effect of PBS (vehicle) or GA injected into the left carotid artery during reperfusion (**Figure** **2**A–C). In short, a catheter was placed in the left common carotid artery and then both common carotid arteries were occluded with atraumatic clips. After 7.5 min, the clips were removed (reperfusion) and 50 µL of 120 × 10^−3^ m GA (*n* = 7 mice) or PBS (0.01 m, *n* = 9) was injected into the left common carotid artery. Sham‐operated mice (*n* = 16) underwent the same surgical procedure without carotid clipping. GCI resulted in neuronal death in the hippocampal cornu ammonis 1 (CA1) region of both hemispheres in the vehicle group (sham vs vehicle, right side, mean difference = 32.78, *p* < 0.001; sham vs vehicle, left side, mean difference = 27.67, *p* < 0.01; one‐way ANOVA followed by Dunnett's multiple comparisons test; Figure 2D). Interestingly, the injection of GA into the left carotid artery during reperfusion resulted in a significant increase in neuronal survival in the left CA1 compared with the contralateral (right) CA1, where a substantial loss of neurons was observed when compared with sham animals (sham vs GA, right side, mean difference = 45.71, *p* < 0.0001). Remarkably, the number of surviving neurons in the left CA1 (ipsilateral to the GA injection) was not different from the sham group (sham vs GA, left side, mean difference = 7.429, *p* > 0.05).

### 2.3 Intraperitoneal GA Treatment during Reperfusion Improves Histopathological and Functional Outcome in the MCAO Mouse Model

**Figure 3:**
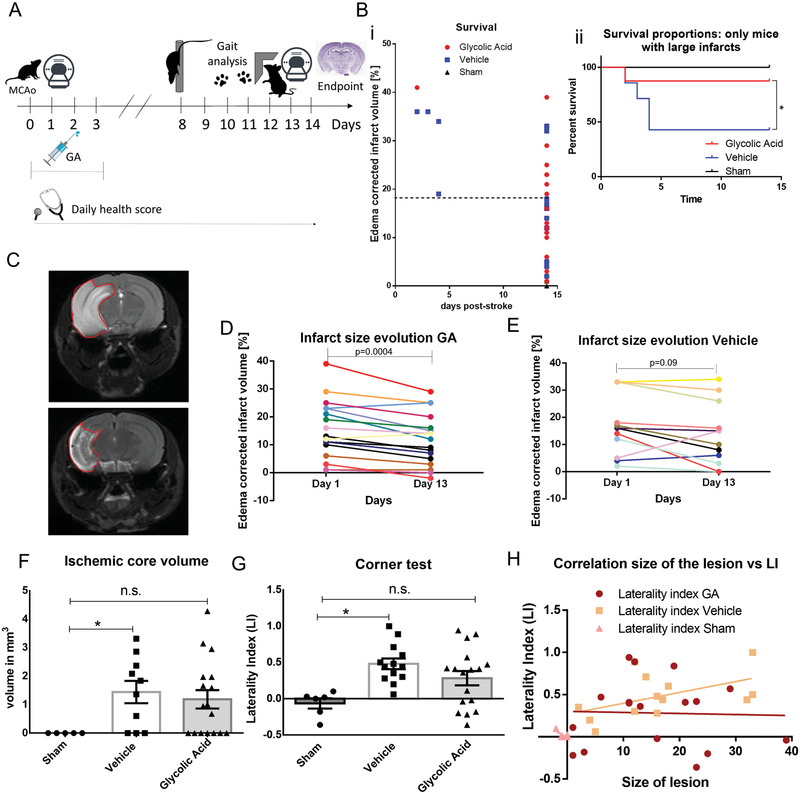
Glycolic acid improves histological and functional outcomes after middle cerebral artery occlusion. A) Experimental design: day 0, middle cerebral artery occlusion (MCAO) or sham operation; day 1, measurement of early infarct size by magnetic resonance imaging (MRI); day 8, motor assessment with the pole test; day 10, gait analysis with the catwalk test; day 12, assessment of laterality (preference for nonaffected side) with the corner test; day 13, measurement of late infarct size by magnetic resonance imaging (MRI); and day 14, brain fixation for further analysis. B‐i) Survival until poststroke day 14. Euthanasia because of fulfilment of humane endpoint criteria was necessary only in mice with infarcts larger than 18% of hemispheric volume. ii) In mice with larger infarcts (>18% of the hemispheric volume), by day 5, 66% of the vehicle group reached the endpoint but only 14.2% of the GA group did (Mantel–Cox survival test: Chi square = 6.215, *p* = 0.0447C; *n*_GA_ = 18, *n*_veh_ = 16, and *n*_sham_ = 6). C) Example MRI image showing the evolution of an MCAO‐induced infarct in a GA‐treated mouse. Upper panel: Day 1 after ischemia. Lower panel: Day 13 after ischemia. D) Evolution of MCAO‐induced infarct in GA‐treated mice: significant reduction of the infarct size on day 13 versus day 1 (paired *t*‐test, *p* = 0.0004, *n*_GA_ = 17). E) Evolution of MCAO‐induced infarct in vehicle‐treated mice: no significant reduction of the infarct size on day 13 versus day 1 (paired *t*‐test, *p* = 0.091, *n*_veh_ = 10. F) Stereological quantification: Although the vehicle group had a significantly higher ischemic core volume than the sham mice (*p* = 0.0243), the difference between GA and sham was not significant (*p* = 0.0638, unpaired *t*‐test; *n*_GA_ = 17, *n*_veh_ = 10, and *n*_sham_ = 5). G) Corner test assessed the preference for the side not affected by ischemia over the affected side, with a higher laterality index (LI) indicating higher impairment of the ischemia‐affected side. The LI was significantly higher in the vehicle‐treated group than in sham mice (*p* = 0.0064). In contrast, the LI of the GA‐treated group was not significantly different from the sham mice (*p* = 0.1042) and the difference between GA‐treated and vehicle‐treated mice was also not significant (*p* = 0.3433, one‐way ANOVA followed by Bonferroni's multiple comparisons test; *n*_GA_ = 17, *n*_veh_ = 13, and *n*_sham_ = 6). H) The functional outcome after MCAO (LI index) was not correlated with the infarct size in GA‐treated mice (Pearson's *r* = −0.03202, *p* = 0.9), but the correlation was almost significant in vehicle‐treated mice (Pearson's *r* = 0.5086, *p* = 0.07; *n*_GA_ = 17, *n*_veh_ = 13, and *n*_sham_ = 6). GA, glycolic acid; LI, laterality index; MCAO, middle cerebral artery occlusion.

To further confirm the observed neuroprotective effects of GA in another relevant in vivo model, we tested the effects of GA in the MCAO mouse model, the most commonly used model in stroke studies.^[^ ^29^ ^]^ The MCAO procedure involves the insertion of a silicon monofilament into the common carotid artery; the monofilament is then advanced until it reaches the origin of the middle cerebral artery (MCA), where it occludes the artery and is left in place for 60 min. After 60 min of MCA occlusion, the monofilament is retracted, and the internal carotid artery is permanently ligated. The sham operation (*n* = 6) uses the identical procedure, but the monofilament is removed as soon as it reaches the origin of the MCA. GA (≈100 µL, 60 mg kg^−1^; *n* = 18) or vehicle (NaCl, ≈100 µL, 0.9%; *n* = 16) was administered by intraperitoneal (i.p.) injection at the end of the operation, immediately after closure of the skin wound. Our experimental timeline is described in detail in **Figure** **3**A. Briefly, MCAO or sham operation was performed on day 0. The next day, the early infarct size was measured by magnetic resonance imaging (MRI). On day 8, mice underwent the first motor function evaluation (pole test). Gait was then assessed on day 10 with the catwalk test, and laterality (preference for nonaffected side), on day 12 with the corner test. On day 13, the late infarct size was measured by MRI, and on day 14, the brain was fixed for further analysis.

As illustrated in Figure S1A in the Supporting Information, the number of surviving mice at the endpoint (14 days after MCAO) was higher in the GA‐treated group than in the vehicle‐treated group, although the difference was not statistically significant (94.44% vs 76.47%, respectively; Mantel–Cox test, Chi square = 3.411, *p* = 0.181); all sham‐operated mice survived until the endpoint. Death occurred only in mice with large infarcts (>18% of the hemispheric volume; Figure 3B‐i). Therefore, we conducted a separate survival analysis in the subgroup with such large infarcts. In this analysis, a Mantel‐Cox test showed that GA significantly increased mouse survival. More specifically, 66% of the vehicle‐treated mice were euthanized by day 5 because they fulfilled humane endpoint criteria, whereas only 14.2% of the GA‐treated mice fulfilled the humane endpoint criteria by that time (Chi square = 6.215, *p* = 0.044; Figure 3B‐ii). Moreover, upon daily careful health monitoring, we observed that GA treatment had no effect on ischemia‐induced weight loss compared with vehicle and showed only a nonsignificant tendency to improve the general health score (evaluation as previously described;^[^ ^30^ ^]^ Figure S1B,C, Supporting Information). The body surface temperature was not influenced by ischemia or treatment (Figure S1D, Supporting Information).

According to our MRI observations, on day 1 after MCAO GA did not significantly alter the total lesion size compared with the vehicle (see Figure S2 in the Supporting Information). However, GA treatment had a strong effect on the evolution of the lesion size. As shown in Figure 3C,D, the infarct size was significantly smaller at day 13 after MCAO than at day 1 (paired *t*‐test, *p* = 0.0004). In contrast, no significant reduction in infarct size was observed in the vehicle group (Figure 3E; paired *t*‐test, *p* = 0.091). This finding shows that GA significantly improved the late outcome after ischemia.

Regarding the assessment of motor function, the pole and catwalk tests did not detect any significant differences between sham‐ and MCAO‐operated mice, regardless of treatment (Figures S3 and S4, and Table S1, Supporting Information). However, GA had a positive effect on performance in the corner test. As shown in Figure 3F, the laterality index (LI) of the corner test was significantly higher after MCAO without treatment than after sham (0.48 ± 0.242 vs −0.065 ± 0.172, respectively; *p* = 0.006; one‐way ANOVA followed by Bonferroni's multiple comparisons test). In contrast, LI in the GA‐treated group was not significantly different from the sham‐operated group (0.26 ± 0.451, *p* = 0.104) (see videos in the Supporting Information). Thus, GA treatment reduced the ischemia‐induced sensorimotor asymmetry to levels similar to those in the sham group. Subsequently, we examined the relationship between infarct size and performance in the corner test. In principle, large infarcts are expected to lead to greater impairments and higher LI values. We found a tendency toward a positive correlation between infarct size and impairment in the corner test in the vehicle‐treated group (Pearson's *r* = 0.508, *p* = 0.075; Figure 3G) but not in the GA‐treated group (Pearson's *r* = −0.032, *p* = 0.902). This finding again suggests that GA improved the functional outcome, especially in animals with larger ischemic lesions.

After the aforementioned in vivo tests, we performed a histological analysis of the brains collected at the endpoint of the experiment on day 14 after MCAO. We used Neurotrace (a Nissl‐based fluorescence staining) to differentiate between healthy and infarcted tissue and NeuN immunostaining to identify and count surviving neurons in infarcted tissue. Nissl substance redistributes within the cell body in injured or regenerating neurons and thereby acts as a marker for the physiological state of the neuron. However, although it identifies damaged areas, it cannot differentiate between the part of the penumbra that survived and is regenerating and the part that has died within the next days after stroke in a patched manner due to apoptosis and did not liquefy and detach. Additionally, during slicing of the brain for histological processing, in some brains with large infarcts we noticed that part of the tissue within the ischemic area had a different, more fragile quality than the surrounding infarct. This part of the tissue tended to detach during histological processing (Figure S5, Supporting Information). We hypothesized that this area may have corresponded to the ischemic core and was undergoing liquefactive necrosis.^[^ ^5^, ^31^, ^32^ ^]^ In this case, one would assume that i) contrary to being an artifact, the volume of missing tissue would show a direct correlation with the ischemic volume measured by MRI and ii) tissue undergoing necrotic liquefication would have a higher water content than the surrounding ischemic tissue and a higher signal in the T2‐weighted MRI scan. Indeed, the volume of missing tissue as measured by stereology was significantly correlated with the infarct volume as measured by MRI on day 13 after MCAO (Figure S6A,B, Supporting Information) in both the vehicle‐treated group (Pearson's *r*_veh_ = 0.598, *p* < 0.01) and the GA‐treated group (Pearson's *r*_GA_ = 0.517, *p* < 0.01). The correlation was significant even when treatment groups were pooled (Pearson's *r* = 0.548, *p* = 0.01). In addition, we performed a detailed assessment of the corresponding MRI images (on day 13) of brains with missing tissue and found that the area that detached during histological processing had a significantly higher contrast in the T2‐weighted MRI scan (i.e., a higher water content) than the surrounding nondetached ischemic area and the contralateral nonaffected hemisphere (one‐way ANOVA, mean difference_detached vs ischemic_ = 35 093 ± 6284, *p* < 0.01; mean difference_detached vs contralateral side_ = 50 576 ± 8623, *p* < 0.01; Figure S6A‐iii, Supporting Information). Taken together, these results support our hypothesis that the missing tissue can be considered as the ischemic core.

Stereological analysis (Stereo Investigator, MBF) provided an estimate of cell density inside the Neurotrace^+^ area and the volume of the infarct (Figure S5, Supporting Information). We observed only a small, nonsignificant reduction in the number of neurons inside the Neurotrace^+^ area, and GA did not change the size of the total Neurotrace+ area (Figure S6A,D, Supporting Information). Although we found a significant difference in the volume of missing tissue (ischemic core) between vehicle and sham (sham, 0.00 ± 0.00; vehicle, 1.444 ± 0.393; *p* = 0.024), we did not find a significant difference between GA and sham (GA, 1.189 ± 0.323; *p* = 0.063) (Figure 3H).

**Figure 4:**
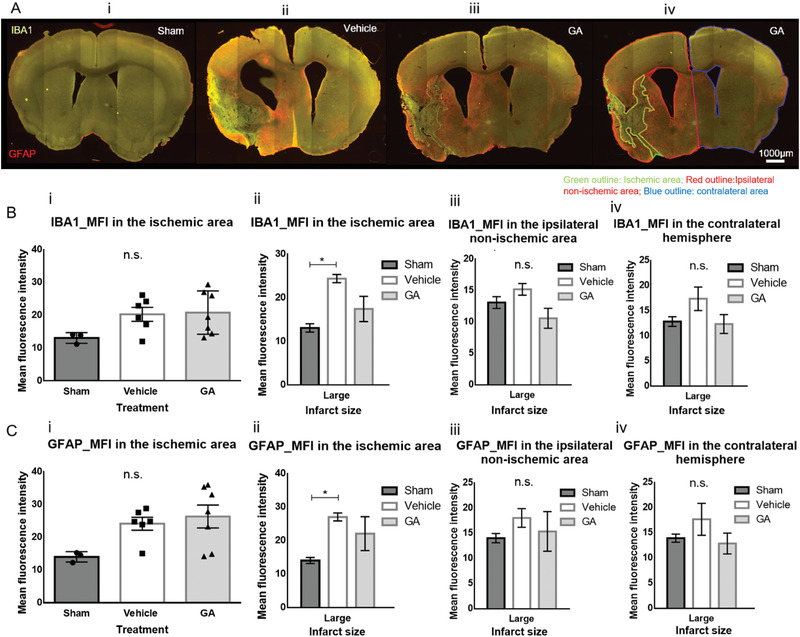
Glycolic acid mitigates the increase in the number of astrocytes and microglia in large infarcts after middle cerebral artery occlusion. A) Fluorescence microscopy images showing the staining for microglia (ionized calcium‐binding adaptor molecule 1 [IBA1], green) and astrocytes (glial fibrillary acidic protein [GFAP], red) in i) a sham brain section, ii) a vehicle‐treated brain section, and iii) a glycolic acid (GA)‐treated brain section with large infarcts. iv) The same brain section as in (iii) indicating the outlines of the different areas in which mean fluorescence intensity (MFI) was measured. B‐i) MFI of IBA1 staining in the ischemic area showing no significant differences between groups (*p* = 0.1482). ii) When large infarcts (>18% of hemispheric volume) were analyzed separately, the MFI of IBA1 inside the infarct area was significantly higher in the vehicle group than in sham mice (mean difference = −11.31, *p* < 0.05), but GA did not differ from sham (mean difference = −4.354, *p* > 0.05). iii) The MFI of IBA1 in the affected (ipsilateral) hemisphere outside the ischemic area was not significantly affected by MCAO (*p* = 0.1032). iv) A trend for higher IBA1 MFI in the contralateral hemisphere was observed in the vehicle group compared with the sham mice and almost reached statistical significance (*p* = 0.0555). C‐i) MFI of GFAP staining in the ischemic area showing no significant differences between groups (*p* = 0.0665). ii) When large infarcts (>18% of hemispheric volume) were analyzed separately, the MFI of GFAP inside the infarct area was significantly higher in the vehicle group than in the sham mice (mean difference = −13.00, *p* < 0.05), but GA did not differ from sham (mean difference = −4.354, *p* > 0.05). The MFI of GFAP in the affected (ipsilateral) hemisphere iii) outside the ischemic area or iv) in the contralateral hemisphere was not significantly affected by MCAO (*p* = 0.6747 and *p* = 0.3412, respectively). For all aforementioned comparisons, one‐way ANOVA followed by Tukey's multiple comparisons test was performed. Data are shown as mean ± SEM; *n*_sham_ = 3, *n*_veh_ = 6, and *n*_GA_ = 7; scale bar: ≈1000 µm. GA, glycolic acid; GFAP, glial fibrillary acidic protein; IBA1, ionized calcium‐binding adaptor molecule 1; MFI, mean fluorescence intensity.

Last, we tested the effect of GA on the MCAO‐associated increase in numbers of astrocytes and microglia in the brain. We used immunohistochemistry to stain for ionized calcium‐binding adaptor molecule 1 (IBA1), a well‐established marker for microglia and macrophages,^[^ ^33^ ^]^ and glial fibrillary acidic protein (GFAP), a well‐established marker for astrocytes^[^ ^34^ ^]^ (**Figure** **4**A‐i–iii). We measured the fluorescence intensity of these markers in the ischemic area, the nonischemic area of the affected hemisphere (ipsilateral), and the nonaffected (contralateral) hemisphere (Figure 4A‐iv). As shown in Figure 4B‐i,C‐i, when the brains were pooled independently of their infarct volume as measured by MRI we observed no significant changes in the signal intensity of microglia or astrocytes inside the ischemic area between sham, vehicle‐ or GA‐treated mice (one‐way ANOVA with Tukey's multiple comparisons test; sham vs vehicle, *p* = 0.148; sham vs GA, *p* = 0.066). When we examined mice with large infarcts separately (infarct volume >18% of the hemispheric volume), the signal intensity of microglia and astrocytes in the ischemic area were significantly higher in the vehicle group than in the sham group (IBA1, mean difference = −11.31, *p* < 0.05; GAFP, mean difference = −13.00, *p* < 0.05). Furthermore, the numbers of microglia and astrocytes in the ischemic area in GA‐treated mice were not significantly different from sham (IBA1, mean difference = −4.35, *p* > 0.05; GFAP, mean difference = −4.354, *p* > 0.05), as shown in Figure 4B‐ii,C‐ii. In the ipsilateral, nonischemic tissue, we found no significant differences in the IBA1 and GFAP fluorescence signal between groups (one‐way ANOVA with Tukey's multiple comparisons test; IBA1, *F* = 3.198, *p* = 0.103; GFAP, *F* = 0.416, *p* = 0.674). In the hemisphere contralateral to the MCAO side, we found a trend for a higher IBA1 signal in the vehicle group compared with the sham group that was close to reaching statistical significance (mean difference = −4.531, *p* = 0.055), whereas the levels in the GA group were similar to sham (mean difference = 0.495, *p* = 0.842). The contralateral GFAP signal did not seem to be significantly affected by MCAO or treatment (*F* = 1.259, *p* = 0.341).

To reduce the time between reperfusion and GA treatment, we performed a second batch of experiments with 30 additional animals. In these experiments, the operations were performed by a different experimenter and, to reduce the time window between reperfusion and treatment, GA was administered via intraperitoneal injection immediately after monofilament removal before suturing the wound. In this set of experiments, no significant difference was observed between groups in any of the parameters analyzed (infarct size, survival, functional tests, general health; see Figures S8–S10 in the Supporting Information), so we could not perform any comparisons between GA and vehicle to test for neuroprotection.

### 2.4 Glycolic Acid Protects the Penumbra and Part of the Core when Administered Intra‐Arterially after Reperfusion in Swine

**Figure 5:**
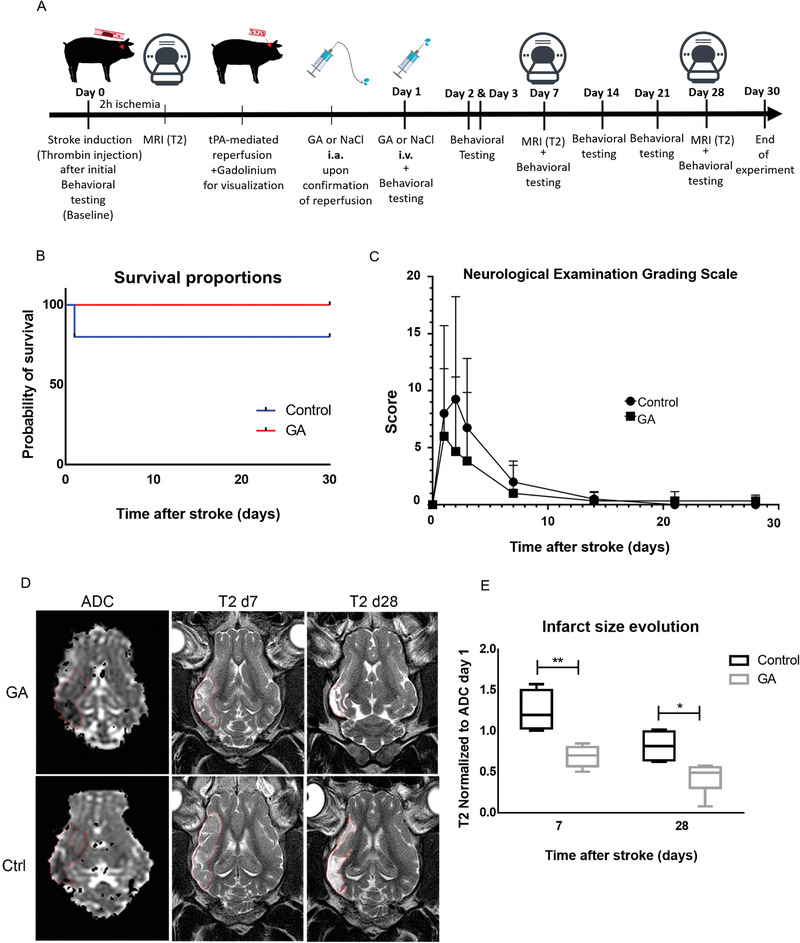
Effect of intra‐arterial administration of glycolic acid on the endovascular model of stroke in swine. A) Experimental design: day 0, behavioral testing to establish baseline function followed by induction of stroke, apparent diffusion coefficient (ADC) magnetic resonance imaging (MRI) 2 h later to assess early infarct size, i.a. tissue plasminogen activator (tPA) injection for reperfusion, and i.a. glycolic acid (GA) or NaCl administration; day 1, GA or NaCl intravenous injection 24 h after stroke; day 1, day 2, day 3, day 7, day 14, day 21, and day 28 after stroke, behavioral testing to evaluate functional outcome; day 7 and day 28 after stroke, T2 MRI to evaluate infarct size evolution; and day 30 after stroke, transcardial perfusion. B) Graph showing the survival proportions in the GA‐treated versus control group. Survival was 100% after GA treatment but only 80% in the control group. C) Scoring of functional outcomes with the Neurological Examination Grading Scale, assessed on day 1, day 2, day 3, day 7, day 14, day 21, and day 28 after stroke. The lower score in the GA group compared with the control group was not statistically significant. *n*_control_ = 5 and *n*_GA_ = 6. D) Representative image showing the ADC signal on day 0 (left column) and the T2 signal on day 7 (middle column) and day 28 (right column) for a GA‐treated (upper row) and control (lower row). E) Quantification of the infarct size. T2 signal on day 7 and day 28 after stroke normalized to ADC on day 0: GA‐treated animals exhibited a significantly higher reduction in the infarct size compared with control on both day 7 and day 28 after stroke (day 7: control, 1.24 ± 0.24; GA, 0.69 ± 0.13, *p* = 0.0016; day 28: control, 0.82 ± 0.19; GA, 0.43 ± 0.18, *p* = 0.0119, unpaired *t*‐test). ADC, apparent diffusion coefficient; GA, glycolic acid; MRI, magnetic resonance imaging.

On the basis of the above results and the differences observed between i.p. and i.a. administration, we hypothesized that i.a. administration of GA at the site of ischemia should lead to better protection against stroke than i.p. administration. Moreover, rodent brains lack the complexity of those of larger mammals such as swine, monkeys, and humans, limiting the translational potential of the results obtained in mice.^[^ ^35^, ^36^ ^]^ To address both concerns, we tested GA in a novel endovascular model of stroke in swine that was recently established by Golubczyk and colleagues and allows for intra‐arterial application of drugs upon pharmacologically induced reperfusion.^[^ ^37^ ^]^ As shown in the experimental design (**Figure** **5**A), after baseline MRI scans ischemia was induced by i.a. injection of 200 U mL^−1^ thrombin. The MRI sequences used were as follows: T2w for anatomical reference, T1 and T1+contrast, perfusion‐weighted imaging (PWI), susceptibility weighted imaging (SWI) to detect thrombus, and diffusion‐weighted imaging (DWI), as well as mean transit time (MTT) and apparent diffusion coefficient (ADC) 2 h after thrombin injection to visualize the infarct core and penumbra (mismatch). After the MRI evaluation of the presence and size of the lesion, 20 mg of tissue plasminogen activator (tPA, 1 mg mL^−1^) was injected into the ascending pharyngeal artery to dissolve the clots, and reperfusion was confirmed by MRI. Once reopening of the occluded vessels was confirmed (≈5–10 min after tPA injection), an 8 mL bolus of GA or vehicle (NaCl) was administered by i.a. injection directly into the reperfused brain territory. The next day, GA (60 mg kg^−1^) or control (NaCl) was injected intravenously. Behavioral testing (Neurological Evaluation Grading Scale)^[^ ^38^ ^]^ was performed before stroke as a baseline and on day 1, day 2, day 3, day 7, day 14, day 21, and day 28 after stroke to assess functional outcome after ischemia. T2 MRI was repeated on day 7 and day 28 to evaluate the infarct size at different stages (see Figure 5A). Animals were euthanized on day 30 after stroke.

Our results showed that, before reperfusion, the infarct size after 2 h of ischemia was not significantly different between groups (see Figure S11 in the Supporting Information). Remarkably, GA treatment had a strong protective effect on both the penumbra and the ischemic core: Whereas the mean ratio of T2 lesion volume on day 7 to ADC lesion volume on day 0 was 1.24 ± 0.24 in the control group, indicating an expansion of the ischemic lesion into the penumbra, it was 0.69 ± 0.13 in the GA‐treated group (control vs GA, *p* = 0.0016), suggesting that GA treatment not only completely protected the penumbra but also reduced the size of the ischemic core (Figure 5C,D). The MRI performed on day 28 after stroke showed a reduction of the lesion size in the control group (T2 Day 28 to ADC Day 0 ratio, 0.82 ± 0.19) and a greater decrease in the GA‐treated group (T2 Day 28 to ADC Day 0 ratio, 0.43 ± 0.18; control vs GA, *p* = 0.0119). We also found a nonsignificant positive effect of GA treatment on survival compared with controls (100% of GA‐treated vs 80% vehicle‐treated mice survived, as shown in Figure 5B).

The score of the neurological evaluation showed no statistically significant difference in behavioral outcome between the experimental and control group at any time point. In absolute values, the difference between the means was highest at day 2, when the score of the neurological evaluation in the GA‐treated group was almost half of that in the control group. This lack of statistical significance could be attributed to the high variability of outcomes between animals. However, the effect size was large (*g* = 0.61) according to Cohen's rule of thumb, indicating that the findings may still be of practical significance. Notably, all animals had almost recovered by day 14, according to a selected battery of behavioral tests, despite the persisting brain damage seen in the MRI, indicating a relatively low sensitivity of the neurological evaluation scale.

## 3 Discussion

The protective role of GA has been studied in only one study in one neurological disease (Parkinson´s disease).^[^ ^19^ ^]^ The study revealed that GA increased the viability of dopaminergic neurons exposed to a neurotoxin in vitro and that the increase in viability was mediated through support of mitochondrial activity.^[^ ^19^ ^]^ The current study is the first to investigate the neuroprotective effects of GA in stroke models both in vitro and in vivo. Important indications that GA may improve stroke outcomes were obtained from previous research: GA was found to protect mitochondrial function, and the genes responsible for the endogenic production of GA (i.e., *djr1.1*, *djr1.2*, and *glod‐4*, which encode glyoxalases) were found to be upregulated in *C. elegans* dauer larva (an evolutionary survival strategy of that enables the larva to survive desiccation/rehydration). *C. elegans* L1 larvae enter dauer, a diapause larval stage, in metabolically unfavorable environmental conditions.^[^ ^14^, ^15^, ^19^ ^]^ Diapause allows invertebrates and nonmammalian vertebrates to survive for extended periods under adverse environmental conditions,^[^ ^39^ ^]^ whereas in mammals, it leads to delayed implantation.^[^ ^40^ ^]^ Broad comparisons of transcriptomic profiles among diapausing stages of distantly related invertebrates suggest that physiologically similar dormancy responses are achieved convergently by diverse regulatory strategies^[^ ^41^ ^]^ or, conversely, that diapause may be phylogenetically conserved, at least among mammals, given the common regulatory factors in different mammalian orders.^[^ ^42^ ^]^ Higher organisms, including humans, produce small amounts of GA in their cells through glyoxalases.^[^ ^21^ ^]^ However, such organisms appear to have lost the ability to dramatically increase the expression of those genes responsible for the production of GA during metabolic stress.^[^ ^43^ ^]^ Supporting this hypothesis, several studies have shown a neuroprotective effect of upregulating DJ‐1 in certain neurological diseases.^[^ ^44^, ^45^, ^46^ ^]^ Although DJ‐1 may appear to be a therapeutic target, gene‐targeting treatments upregulating DJ‐1 would need to be performed at least 24 h before an ischemic insult; clearly, such an approach cannot be applied in clinical care, as it would be required that the time‐point of the ischemic insult is known in advance. In addition, it is unclear whether large enough amounts of glyoxal and methylglyoxal, the substrates of DJ‐1 and glyoxalase domain‐containing protein 4 (GLOD‐4), are produced during ischemia and reperfusion to obtain the high amounts of GA and DL needed to allow them to exert their protective effect. Therefore, we hypothesized that artificially increasing GA levels in the brain would protect tissue from damage in the hypoxic state and metabolic halt induced by an ischemic stroke.

In order to test our hypothesis, we investigated the effects of GA in in vitro (OGD) and in vivo models of ischemia/reperfusion (GCI and MCAO). Our in vitro experiments showed that treatment with GA during reperfusion (i.e., during the change from ischemic to normal medium) resulted in strong protection against OGD‐induced necrotic and apoptotic neuronal death. To differentiate between both types of cell death, we measured PI and NeuN 30 min after reperfusion to quantify necrotic cells and stained against NeuN and DRAQ5 to measure total neuronal loss 72 h after OGD to quantify apoptosis. PI is widely used to detect necrosis.^[^ ^47^, ^48^, ^49^, ^50^ ^]^ The difference in the total amount of neurons 72 h after OGD allows one to discriminate between necrotic and apoptotic death.

Glutamate‐mediated excitotoxicity has been shown to play a role in stroke and ischemia–reperfusion damage through increases in [Ca^2+^]_i_, which has a negative impact on cellular function and can activate apoptosis. In a recent study, we showed that GA reduces [Ca^2+^]_i_ in HeLa cells and sperm cells and enhances mitochondrial energy production.^[^ ^25^ ^]^ Therefore, we tested whether it also had such an effect on [Ca^2+^]_i_ in cortical neurons in the absence and presence of glutamate. Indeed, our results showed that increasing concentrations of GA reduced [Ca^2+^]_i_ in a concentration‐dependent manner in both the absence and presence of 300 × 10^−6^ m glutamate, suggesting that the reduction of [Ca^2+^]_i_ could be one of the underlying mechanisms of action for the protective effect of GA in stroke.

We confirmed these strong neuroprotective properties of GA in 2 mouse models and an endovascular swine model of stroke. In general, postischemia treatment by i.a. administration of GA into the left carotid (GCI mice) or ipsilateral ascending pharyngeal artery (swine) immediately after reperfusion had a strong, positive effect on neuronal survival; the effect was not as strong after i.p. administration of GA in MCAO mice. In addition, groups treated with GA exhibited a significant reduction in infarct volume (MCAO and swine model) and complete rescue of neurons in brain areas with anoxic/hypoxic damage (GCI model). These effects were correlated with a better, if not significant, functional outcome and, in the case of the MCAO model, a nonsignificant reduction of the inflammatory reaction.

In the GCI mouse model, the method used to induce ischemia caused bilateral damage, but GA was applied only to the left carotid artery. In this way, the side ipsilateral to the intra‐arterial GA injection was considered as the “treated side.” In contrast, the opposite hemisphere was regarded as the “control side” in each animal. The lack of effect on the contralateral side indicates that choosing an injection site of high proximity as close as possible to the damaged brain tissue can lead to better results because of faster distribution and increased accumulation of GA. This finding is in accordance with previous studies, which showed that the i.a. application route is more efficient than other methods in yielding high concentrations of the administered substance within a region of interest.^[^ ^51^, ^52^, ^53^ ^]^ Research has also shown that the permeability of the blood‐brain barrier increases after a stroke^[^ ^54^ ^]^ and that i.a. injection of hyperosmotic solutions (such as the high concentrations of GA in our study) increases the permeability of the blood‐brain barrier 30‐ to 50‐fold for a short but essential time window (≈6 min).^[^ ^51^ ^]^ Thus, we hypothesized that ipsilateral intracarotid administration of GA may also positively influence the histological and functional outcome in focal stroke.

To examine that hypothesis, we aimed to administer GA intra‐arterially in a mouse model of focal stroke. We selected the most commonly used and reliable animal model of stroke, the MCAO mouse model. However, because of technical difficulties, intracarotid application was not feasible in the available setting. Therefore, we decided to test the effects of i.p. GA on the outcome of focal ischemia. In agreement with previous findings in this model, MCAO led to infarcts that included large parts of the ipsilateral cortex and striatum, as observed by MRI (Figure 3C). Independent of the treatment, the infarct size showed a relatively high variability (Figure S3, Supporting Information), ranging from 2% to 40% of the ipsilateral hemisphere volume, although the mean was ≈15%, which is similar to previous reports.^[^ ^55^ ^]^ Large variations in ischemic volumes are not uncommon in this animal model and are generally attributed to the cerebral vasculature of C57Bl6 mice. Up to 40% variability is considered acceptable, as determined by a published protocol of standard operating procedures.^[^ ^56^ ^]^ This variability is due to interindividual anatomical differences in the Circle of Willis, i.e., the presence or absence of the ipsilateral posterior communicating artery (PcomA).^[^ ^57^, ^58^ ^]^ To avoid this high interindividual variability, we used MRI to compare the intraindividual infarct volume reduction between day 1 and day 13 after ischemia with a paired *t*‐test and found that GA but not vehicle significantly improved the evolution of infarct volume in this period (Figure 3D,E). Thus, GA treatment resulted in a better long‐term anatomical outcome. GA treatment also positively affected MCAO‐induced mortality and functional outcome and did not show any signs of toxicity. Furthermore, when considering only infarcts with volumes greater than 18% of the hemispheric volume, GA reduced mortality compared with vehicle treatment.

Regarding functional outcome, the corner test detected a positive effect of GA treatment on sensorimotor function after MCAO. Our results showed that vehicle‐treated mice displayed significantly lower performance than sham‐operated mice and that i.p. administration of GA reduced sensorimotor asymmetry to levels that were not significantly different from sham (Figure 3F). The corner test has consistently and reliably detected the influence of various substances on functional outcome after MCAO in previous studies^[^ ^59^, ^60^, ^61^ ^]^ and was the only functional test used in the present study that was sensitive enough to detect differences between sham‐ and vehicle‐treated mice. Interestingly, the improvement in performance achieved by GA was independent of the infarct volume, suggesting that GA either increased the number of surviving neurons inside this area or supported neuronal plasticity. The quantification of surviving neurons within the ischemic area did not show any significant differences between any of the groups. There are two possible hypotheses that may explain this surprising result: i) the Neurotrace^+^ area corresponds to the penumbra and ii) NeuN protein is expressed not only by neurons but also by astrocytes^[^ ^62^, ^63^ ^]^ (astrocytes are known to be increased after stroke in the infarct and peri‐infarct area^[^ ^64^, ^65^, ^66^ ^]^). Supporting the first hypothesis, at the MRI on day 14, we found a significant positive correlation between ischemic volume and both the volume of missing tissue and the increased T2 signal in the areas of missing tissue compared with adjacent areas. This finding suggests that these areas had a higher water content because unrelated artifacts would not have shown any correlation with any of these parameters, and it may reflect an underlying liquefactive transformation on day 14 after stroke, as previously described.^[^ ^31^, ^32^ ^]^ This missing tissue should not be mistaken for an enlargement of the lateral ventricles. In this study, we observed missing tissue even in regions posterior to the end of the lateral ventricles (approximately at the bregma between −3.7 and −3.15 mm). In this region, the pyramidal cell layer and the CA1 region of the hippocampus were completely missing (example in Figure S5D in the Supporting Information). If this were the case, the remaining Neurotrace^+^ tissue around this area would correspond to the penumbra. In the penumbra, the neuronal loss is patchy, as described previously,^[^ ^67^, ^68^ ^]^ and therefore it is difficult to detect a significant reduction in cell density. Although GA did not alter the cell density in the infarct site, it reduced the ischemic core volume (measured as the missing volume) compared with the vehicle (the GA group was not significantly different from sham) and thus contributed to the improved functional outcome observed after stroke. Nevertheless, the hypothesis that this lack of difference is due to the presence of NeuN^+^ astrocytes or that the functional improvement is related to an increase in neuronal plasticity cannot be excluded and should be further investigated.

Because ischemia induces a robust immune response in the human and mouse brain, including an increase in numbers of astrocytes and microglia,^[^ ^62^ ^]^ we also tested whether GA affected the immune reaction in this model. When we evaluated the fluorescence marker levels of astrocytes and microglia between vehicle‐ and GA‐treated groups independently of the infarct volume, we detected no significant difference. However, as indicated by functional and survival data, when analyzed independently, larger infarcts were associated with a significantly higher GFAP and Iba1 signal in the vehicle group but not in the GA group compared with sham. We did not observe this difference in smaller infarcts, where GFAP and Iba1 signals were increased in the both the vehicle‐ and GA‐treated mice.

A possible explanation for the differences in the results between small and large infarct volumes may be the regions affected in each case. In brains with smaller infarct volumes, the main affected area was the striatum, which is the primary target of MCA supply and is known to have very low collateral density.^[^ ^69^, ^70^, ^71^ ^]^ The striatum is consistently infarcted even in mild strokes (30 min MCAO),^[^ ^29^, ^72^ ^]^ whereas in severe MCAO (lasting 120 min), the necrotic area expands over the striatum to a significant fraction of the surrounding cortex, where a penumbra area is observed because of better collateral perfusion.^[^ ^73^, ^74^ ^]^ Moreover, after MCAO the striatum displays certain metabolic changes that are typical of the infarct core rather than the penumbral area.^[^ ^71^ ^]^

Comparing the GCI and MCAO models, the latter did not show significant differences between GA and vehicle groups, despite the apparent improvement in the infarct core and corner test performance for the GA group, which was not significantly different to sham. We believe that this is due to the route of administration in the MCAO model that delays the timing and reduces the concentration of GA achieved in the brain after reperfusion. Moreover, the other functional tests implemented in this model (pole test, catwalk test) were not sensitive enough to detect any significant differences between sham and MCAO independently of the treatment, so the lack of significant differences between the GA and vehicle groups was not unexpected.

Finally, rodent models lack the complexity of brains in larger mammals such as monkeys, swine, or humans, which have lobes, gyri, and sulci and much longer and more numerous axons. These differences may limit the potential of translating results from the mouse model into clinical practice. Therefore, we decided to test the effects of GA in an endovascular model of ischemic stroke in swine that was recently developed by Golubczyk et al.^[^ ^37^ ^]^ In this animal model, i.a. administration of GA during reperfusion after the pharmacological lysis of a clot showed remarkable results: GA treatment was able not only to completely salvage the penumbra but also to reduce the damage in the ischemic core. This reduction was associated with a nonsignificant improvement in functional outcome. However, in our study the functional data were difficult to interpret because large infarcts caused the pigs to lie on the ground, and they subsequently either recovered and stood up or, as in the case of one vehicle‐treated animal, died. The fact that even swine with large infarcts could completely recover within a few days might reflect a lack of sensitivity of the functional tests performed. The lack of statistical significance could also be attributed to the high variability of outcomes between animals and the low number of animals used. In our experimental setup, the use of each animal as its own control (by MRI evaluation of damage before and after GA administration) was highly advantageous and relevant to the clinical setting. However, this approach was not possible for the functional tests, which reduced the number of animals in each group although it should be closer to a typical clinical study. As an example, a group size of at least 35 was necessary to show significant differences in neurological outcome with the same neurological score,^[^ ^38^ ^]^ but a study with such a large sample size would be impossible to perform in our setting. Despite the lack of statistical significance in our results on functional outcome, we believe that the behavioral results add value to the study. In absolute numbers, on day 2 the behavioral deficit in the control group was nearly twice that in the GA‐treated group and the effect size (*g* = 0.61) was large according to Cohen's rule of thumb, indicating that the results may still have a practical significance. In any case, this endovascular model of ischemic stroke in swine, an animal in which the anatomical brain complexity is comparable to that in humans, almost completely mimics the ischemia time and current protocols used in stroke units. In the clinical setting, to protect the brain from the negative consequences of reperfusion, GA could be administered intra‐arterially immediately after mechanical thrombectomy or localized pharmacological thrombolysis by using the same catheter that is normally used to perform these procedures. It is known that the mean time between patient arrival and thrombectomy/thrombolysis is 2.5 h,^[^ ^8^ ^]^ which is a relatively small deviation from the administration protocol used in the swine model (2 h of ischemia + ≈15 min until reperfusion was observed). Thus, we believe that GA treatment is particularly valuable in the current era of mechanical thrombectomy and local lysis, and we hypothesize that it can facilitate broadening indications for these two procedures; however, this hypothesis can be tested only in clinical settings. To sum up, we believe that the results obtained in this model are representative of the results that can be expected in stroke patients.

The underlying molecular mechanism by which GA exerts its neuroprotective effects is currently being investigated in an ongoing study by our group. Previous research has suggested that GA can act as a calcium chelator.^[^ ^75^ ^]^ We recently published data suggesting that GA reduces [Ca^2+^]_i_, which is increased during stroke‐induced excitotoxicity and preserves normal mitochondrial function, which is impaired after stroke.^[^ ^25^ ^]^ Our in vitro data obtained from cortical neurons suggest that the reduction of [Ca^2+^]_i_ is the critical neuroprotective factor in this case, and we hypothesize that this is the case because—as shown in the aforementioned study—increases in energy production were observed with GA concentrations as low as 2.5 × 10^−3^ m. Nevertheless, this concentration was not as effective as higher concentrations in preventing neuronal death. Regulation of [Ca^2+^]_i_ is a common feature among species that undergo desiccation at any developmental stage. The most extreme form of vertebrate diapause occurs in annual killifish, a polyphyletic assemblage of freshwater fish within the Aplocheilidae family (order Cyprinodontiformes), which are able to complete their life cycle in ephemeral habitats within a year.^[^ ^76^, ^77^, ^78^, ^79^, ^80^ ^]^ When their pond dries up, adult killifish die, leaving their fertilized eggs buried in the substrate, and developing embryos survive until the following wet season by entering diapause.^[^ ^81^ ^]^ Interestingly, in this species many of the genes that are upregulated only during desiccation in the diapause stage are linked to the regulation of calcium.^[^ ^82^ ^]^

## 4 Conclusion

Taken together, our results suggest that GA treatment has a solid neuroprotective effect when administered at high concentrations, especially intra‐arterially, immediately after the ischemic insult. This effect is mediated by the reduction of [Ca^2+^]_i_ during excitotoxicity. However, our results in mice have certain limitations, for example i) all experiments were performed on young male mice, so the effect in female mice and/or different age groups still needs to be studied, ii) mouse strains (C57BL/6N vs C57BL/6J), ischemia duration, analysis time points, and treatment administration routes differed between the two in vivo models, and this can create a discrepancy in their sensitivity, iii) although both models mimicked the mechanical thrombectomy that occurs in the clinical setting, neither of them mimicked the pharmacological thrombolysis that is achieved clinically with recombinant tissue plasminogen activator (rTPA), which could interact with GA.^[^ ^37^, ^83^, ^84^ ^]^ A swine model of stroke overcame these limitations by allowing thrombolysis with TPA and the i.a. delivery of GA. The results in this model not only further validated the results in the mouse models but even exceeded the expected outcome as GA ultimately protected the penumbra and even reduced the ischemic core. These results challenge the current knowledge that the fate of the ischemic core, as measured by DWI‐sequences in MRI, is already determined and could have important implications in the clinical setting as a potential treatment for stroke patients.

## 5 Experimental Section

### In Vitro Hypoxia—OGD Procedure

To simulate IR in vitro, primary cortical neurons from E15.5 mice embryos were prepared and plated in 2 × 96‐well plates (one for normoxia and one for evaluation of necrosis with DRAQ5 and PI 30 min after OGD or, alternatively, one for normoxia and one for evaluation of survival with NeuN 72 h after OGD). Plates were coated with poly‐l‐lysine (PLL), 10 µg per well in 100 µL H_2_0, and neurons were plated at a concentration of 1 × 10^6^ cells mL^−1^ in Neurobasal A (#1088022) media containing 5% B27 (#17504044), Pen‐Strep (#10378016), and l‐glutamine (#25030024) (all from Thermo Fischer Scientific, MA, USA), as previously described.^[^ ^85^ ^]^ On DIV 7, neurons were placed within the anoxic atmosphere and incubated in an N_2_‐filled gas chamber for 1 h with glucose‐free acidic anoxic buffer (previously deoxygenated in an autoclave and bubbled with N_2_ for 20 min) containing (in mmol L^−1^): 140 NaCl, 3.6 KCl, 1.2 MgSO_4_, 1 CaCl_2_, and 20 HEPES at pH 6.4 and supplemented with 4 µmol L^−1^ resazurin, 100 µmol L^−1^ ascorbic acid, 0.5 mmol L^−1^ dithionite, and 100 U mL^−1^ superoxide dismutase. To mimic reperfusion, the anoxic buffer was washed out and replaced with Neurobasal A media as above but without phenol red and without antioxidants (pH 7.4) and supplemented with GA (stock solution 3.92 m diluted in water, pH adjusted to 7 with NaOH, final concentration 10 × 10^−3^ and 20 × 10^−3^ m, Sigma‐Aldrich, Germany) or vehicle (ddH_2_O). For the normoxic group, media was replaced with Neurobasal A media without phenol red and without antioxidants, and neurons were kept at 5% CO_2_ and 37 °C. The reperfusion media in the plates used to evaluate necrosis included PI (81845, end concentration 5 × 10^−6^ m, Sigma‐Aldrich, Germany), and these plates were fixed with 2% paraformaldehyde (PFA) 30 min after the OGD/normoxia. Plates used to evaluate apoptosis after 72 h were kept at 5% CO_2_ and 37 °C for 1 h, after which more medium was added to the well to achieve a concentration of 5 × 10^−3^ m GA and the plates were left in the incubator for 72 h until fixation. This approach ensured that neurons were subjected to the same OGD conditions.

### In Vitro Hypoxia—Cell Immunofluorescence and Quantification

To evaluate necrosis, cells were fixed as mentioned above 30 min after OGD/normoxia and imaged with an Opera High‐Content Screening System (20× Air, PI: Ex: 561, Em: 600/40, PerkinElmer, MA, USA). Cells were then incubated at room temperature (RT) with blocking solution (5% donkey serum, 0.05% Triton X‐100, and PBS) for 1 h. Primary antibody (NeuN Polyclonal, ab104224, Abcam, MA, USA) was added in block solution (1:1000 dilution) and incubated for 1 h at RT and then overnight at 4 °C. The next day, cells were washed three times (for 10 min per wash) with PBS and then incubated with the secondary antibody solution (AlexaFluor 555 a31570, Thermo Fisher Scientific, MA, USA; 1:500 in blocking buffer) for 2 h at RT and protected from light. After three washings with PBS, the cells were ready for observation. Microscopy images were obtained with an Opera High‐Content Screening System (20× Air, Ex: 488 nm, Em: 568, PerkinElmer, MA USA). The same 10 fields per well were imaged after addition of PI and after staining with NeuN, and the numbers of NeuN^+^ and PI^+^ neurons were quantified with Image J software (National Institutes of Health, Bethesda, MD) in a semiautomated manner.

To quantify the surviving neurons, at 72 h after OGD/normoxia cells were fixed with 2% PFA for 35 min at RT or overnight at 4 °C. Cells were then incubated at RT with blocking solution (5% donkey serum, 0.05% Triton X‐100, and PBS) for 1 h. Primary antibody (NeuN Polyclonal, ab104224, Abcam, USA) was added in blocking solution (1:1000 dilution) and incubated for 1 h at RT and then overnight at 4 °C. The next day, cells were washed three times (for 10 min per wash) with PBS and then incubated with the secondary antibody solution (AlexaFluor 555 a31570 Thermo Fisher Scientific, MA, USA; 1:500 in blocking buffer) for 2 h at RT and protected from light. After three washings with PBS, the neurons were incubated with DRAQ5 (62251, 1:10 000, Thermo Fischer Scientific, MA, USA) diluted in PBS for 5 min at RT, followed by aspiration and 3× PBS rinsing. Microscopy images were obtained with an Opera High‐Content Screening System (20× Air, NeuN: Ex: 488 nm, Em: 568, DRAQ5: Ex. 640, Em: 690/50 nm PerkinElmer, MA, USA). Ten fields per well were imaged, and the number of NeuN^+^ neurons was quantified with Image J software (National Institutes of Health, BethesdaMD, USA) in a semiautomated manner.

### In Vitro Intracellular Calcium Measurements on Cortical Neurons

Primary cortical neurons from E15.5 C57Bl6 mouse embryos were prepared and plated in 96‐well plates as previously described. At DIV 7, neurons were incubated for 20 min at 37 °C with 50 µL per well of 2.5 × 10^−6^ m FLUO‐4‐AM (#F14201; Thermo Fischer Scientific, MA, USA) diluted in HBSS. After incubation, FLUO‐4‐AM was replaced by HBSS (H6648, Sigma‐Aldrich, Germany) for 5 min at 37 °C. HBSS was then replaced by 50 µL per well of fresh medium A (Neurobasal A media [#1088022]) containing 2% B27 (#17504044), 0.4% Pen‐Strep (#10378016) and 1% l‐glutamine (#25030024), all from Thermo Fischer Scientific, MA, USA. The cells were left to stabilize in medium A for 10 min before starting the measurements. Fluorescence measurements were performed in a FLUOstar Optima (BMG Labtech, Germany) plate reader with the 485 nm excitation filter and the 520 nm emission filter. A baseline was recorded for 3 min, after which the fluorometer was paused and 5 µL of either PBS (vehicle), 4.5 × 10^−3^ m NaCl (as osmolality control), or 5 × 10^−3^, 10 × 10^−3^, and 20 × 10^−3^ m of GA diluted in PBS were added to each well. The measurement continued for the next 6 min and was followed by another pause for the addition of ionomycin (#I3909; Sigma‐Aldrich, Germany) at a concentration of 2 × 10^−6^ m. The measurement was allowed to continue for a total of 25 cycles (762 s).

For the experiments with glutamate, the baseline was recorded up to the addition of 300 × 10^−6^ m glutamate (#G8415; Sigma‐Aldrich, Germany) after 230 s of measurements and the posterior addition of GA at time‐point 296 s. After that, the measurements continued for a total of 25 cycles without adding ionomycin.

### GCI Mouse Model—Animals and Housing

A total of 32 six‐ to eight‐week‐old male C57BL/6N mice (Charles River Laboratories, Sulzfeld, Germany) were used in this part of the study. All experimental procedures were performed in accordance with German guidelines on animal welfare and were approved by the local regulatory committee (Regierung von Oberbayern, Munich, Germany). The mice were housed at the Institute of Stroke and Dementia Research animal husbandry area, where they were kept under a 12 h light/dark cycle (lights on from 6:00 to 18:00) in an enriched environment with ad libitum access to food and water.

### GCI Mouse Model—Surgery

GCI was induced as previously described.^[^ ^27^, ^28^ ^]^ Briefly, 32 C57BL/6N six‐ to eight‐week‐old male mice with a bodyweight of 20 to 24 g (Charles River Laboratories, Sulzfeld, Germany) were anesthetized with a combination of buprenorphine (0.1 mg kg^−1^, Essex Pharma, Germany) and 2% isoflurane (Halocarbon Laboratories, Peachtree Corners, GA, USA) in 50% O_2_/50% N_2_. Body temperature was tightly controlled with a feedback‐controlled heating pad (FHC, Bowdoinham, ME, USA). Regional cerebral blood flow (rCBF) was continuously monitored over the right hemisphere with a laser Doppler perfusion monitor (Periflux 4001 Master, Perimed, Sweden). The neck was opened, and both common carotid arteries were exposed. A catheter was placed in the left common carotid artery, and then both common carotid arteries were occluded with atraumatic clips. After 7.5 min, the clips were removed and 50 µL of GA (3.92 m stock solution in water, with a pH adjusted to 7 with NaOH; end concentration in PBS, 120 × 10^−3^ m; *n* = 7 mice) or PBS (0.01 m, *n* = 9) were injected into the left common carotid artery. Thereafter, the catheter was removed, the incision was sutured, and the animals received 100 µL of carprofen (1 mg mL^−1^) s.c. for postoperative analgesia. Sham‐operated mice (*n* = 16) underwent the same surgical procedure without carotid clipping. Animals were randomly and blindly allocated to the respective treatment group shortly before injection of GA or PBS.

### GCI Mouse Model—Histology

One week after GCI, mice were reanesthetized and transcardially perfused with 4% PFA. Brains were extracted, dehydrated, embedded in paraffin, cut into 4 µm thick coronal sections and stained with cresyl violet for neuronal cell counting. Intact neurons in the hippocampal CA1a, CA1b, CA2, and CA3 subregions were counted with Image J (National Institutes of Health, Bethesda, MD) by an investigator blinded to the treatment of the mice. For this analysis, sections were assessed per mouse.

### MCAO Mouse Model—Animals and Housing

A total of 40 C57BL/6J 12‐week‐old male mice (Janvier Labs, Le Genest‐Saint‐Isle, France) were used in this study. All experimental procedures were performed according to the ARRIVE guidelines, European Community Council Directives 86/609/EEC, and German national laws and were approved by the local authority (Landesamt für Gesundheit und Soziales, Berlin, Germany). The mice were housed at the Charité animal facility, where they were kept under a 12 h light/dark cycle (lights on from 6:00 to 18:00) in an enriched environment with ad libitum access to food and water.

### MCAO Mouse Model—Surgery

Transient (60 min) MCAO was performed according to a standard protocol.^[^ ^86^ ^]^ This process creates a brain infarction in the MCA area. The resulting infarct includes broad damage in the striatum and ipsilateral cerebral cortex and the presence of a relatively small penumbral area in the cortex.^[^ ^74^ ^]^ A total of 34 mice underwent MCAO surgery (18 treated with GA and 16 with the vehicle), and 6 mice received sham surgery and GA treatment. Animals were randomly allocated to the two treatment MCAO groups or the sham group. Anesthesia was induced with 2.5% isoflurane (Forene, Abbott, Wiesbaden, Germany) in a 1:2 oxygen/nitrous oxide mixture and maintained at 1.0% to 1.5% throughout the operation. A silicon rubber‐coated monofilament (no. 701956PK5Re Doccol Corporation, Sharon, MA, USA) of 0.19 ± 0.01 mm in diameter was inserted into the common carotid artery and advanced until it reached the origin of the MCA, where it remained for 60 min while the mouse was allowed to recover from anesthesia inside a heated cage that maintained body temperature at 37 °C. At the end of the 60 min of MCA occlusion, the mouse was reanesthetized, the monofilament was gently retracted, and the internal carotid artery was permanently ligated. Exactly the same procedure was used in the sham operation, but the monofilament was removed as soon as it reached the origin of the MCA. GA or NaCl i.p. injection was performed at the end of the operation, immediately after closure of the skin wound. Bupivacaine gel (1%) was topically applied to the wound for postsurgical pain prevention, and the mouse received 500 µL of saline subcutaneously for rehydration. After the operation, mice were placed in a heated cage for 1 h before being returned to their home cages. In the recovery phase after the operation, food and pellets soaked in water were provided on the cage floor.

The second batch of MCAO operations was performed to test whether administering GA sooner after MCAO would improve the efficacy of the treatment. This batch included 30 12‐week‐old male C57Bl/6J mice (14 treated with GA, 14 treated with vehicle, and 2 untreated [sham]). In this experiment, the i.p. injection was performed immediately after removal of the monofilament to minimize the delay introduced by wound suturing. Except for the timing of administration of GA or vehicle, the rest of the surgical and postoperation process remained unaltered.

### MCAO Mouse Model—Substance Preparation for i.p. Administration

100 µL of GA or 0.9% NaCl (vehicle) solution were injected i.p. after the operation until day 3 after the MCAO/sham operation. For the GA solution, GA powder (Glycolic Acid, Sigma‐Aldrich) was diluted in pure water to obtain a final concentration of 15.6 mg mL^−1^ (60 mg kg^−1^) for i.p. injection. The pH was adjusted to 7 with NaOH.

### MCAO Mouse Model—Histology

Fourteen days after MCAO, mice were deeply anesthetized with ketamine/xylazine (150 and 15 mg kg^−1^, respectively) and, upon complete loss of pedal reflexes, transcardially perfused with a 0.1 m PBS solution. Brains were carefully extracted and kept in 4% PFA in a 15 mL Falcon tube overnight at 4 °C. On the next day, for cryoprotection brains were incubated in 30% sucrose until they sank. Then, brains were frozen with the *n*‐butanol procedure in which 10 mL of *n*‐butanol were added in a 15 mL Falcon tube and cooled in liquid nitrogen to −50 °C. Once this temperature was reached, the brains were inserted into the Falcon tube, which was kept in liquid nitrogen for one minute to cool it to −80 °C. The brains were then ready for immediate storage at −80 °C. Deeply frozen brain tissue was mounted on the sliding microtome (Leica SM210R) with OCT Tissue‐Tek (Sakura Finetek Europe B.V., NL) and kept frozen with the addition of dry ice for cutting. Sequential sections of 60 µm in thickness were acquired and transferred sequentially into 96‐well plates filled with freezing medium (50% PBS, 25% glycerol, and 25% ethylene glycol) for further storage at −20 °C.

### MCAO Mouse Model—Staining of Brain Sections

NeuroTrace staining was performed for the histological definition of lesion volume, and NeuN staining was performed for quantification of surviving neurons. On staining day 1, sections were washed with PBS (pH 7.4) three times for 10 min each and then incubated with blocking buffer (5% donkey serum, 0.1% TritonX‐100 in PBS) for 1 h at RT. Subsequently, the primary antibody (rabbit anti‐NeuN, ABN78, Sigma‐Aldrich, Germany) was added in a 1:500 dilution and the sections were kept at 4 °C overnight. On staining day 2, after washing three times for 10 min each with 0.1% Triton‐PBS, the sections were incubated with the secondary antibody solution containing 1% donkey serum, 1:500 Ab” (antirabbit Alexa 568, A‐10042 Invitrogen, USA), 1:250 NeuroTrace 435 (N21479 Invitrogen, USA) and 0.1% Triton in PBS. After 2 h at RT, the sections were washed again with PBS three times for 10 min each, mounted on gelatin‐covered glass slides and coverslipped for later observation. For the GFAP and Iba1 staining of brain sections, the same process was followed, but the primary antibodies were goat anti‐GFAP (ab53554, Abcam USA) in 1:750 dilution and rabbit anti‐Iba1 (019‐19741, FUJIFILM Wako Pure Chemical Corporation USA) in 1:750 dilution. The secondary antibodies used were donkey antigoat Alexa 555 (A‐21432 Invitrogen, USA) for the GFAP labeling and donkey antirabbit Alexa 488 (A‐21206, Invitrogen, USA) for the Iba1 labeling.

### MCAO Mouse Model—Functional Tests

Besides causing histological damage, MCAO is known to induce deficits in motor function, including motor coordination, balance, and muscle strength, with mice showing a preference for using the nonaffected limb.^[^ ^87^ ^]^ Therefore, different functional tests were performed to assess such deficits.

### MCAO Mouse Model—Pole Test

The pole test is a method for simple motor function evaluation;^[^ ^88^, ^89^ ^]^ after preoperative training, the mice performed the test on day 8 after MCAO. Animals were placed on top of a vertical pole, 10 mm in diameter and 55 cm long, and were observed as they turned around and descended the pole (snout first). The scoring began when the animal started the turning movement. The time taken to make a full 180° turn (time to turn) and latency to reach the ground (time to descend) were recorded. Mice had to descend successfully three times. Trials in which mice took longer than 5 s to turn or longer than 20 s to descend were excluded. Pausing was also an exclusion criterion.

### MCAO Mouse Model—Gait Analysis

For gait analysis, the CatWalk (Noldus Information Technology) automated, computer‐assisted system was used, which is often used to assess locomotion defects in stroke mouse models.^[^ ^90^ ^]^ The CatWalk apparatus comprises a long, elevated glass runway platform that is fluorescently illuminated from the inside; the light is reflected in the direction of the floor when pressure (weight) is applied on top. A camera is mounted underneath the glass platform to record the walking pattern. At the beginning of the experiment, the animals’ home cage was placed at one end of the platform. Then, the mice were placed on the opposite end and allowed to walk across the platform voluntarily toward their home cage. Analysis was performed with CatWalk XT 10.5 Software, which visualizes the footprints and calculates statistics regarding their dimensions and the time and distance ratios between footfalls. For a trial to be considered successful, the speed should not vary by more than 60% and the run should be uninterrupted (i.e., the mouse should not stop on the runway). Unsuccessful trials were repeated until three successful trials were reached. Before baseline acquisition, mice were preoperatively trained for 3 days with the CatWalk system (3 runs per day). The test was then performed on day 10 after MCAO.

### MCAO Mouse Model—Corner Test

The corner test, which was developed for measuring sensorimotor asymmetries after unilateral corticostriatal damage, was performed on day 12 after stroke.^[^ ^91^ ^]^ The testing arena was composed of two connected cardboard walls that form a corner of ≈30°. A small opening was left at the junction of the walls to motivate the mice to reach deep into the corner. At the beginning of the test, each animal was placed halfway from the corner and facing it. As the mice walk into the corner, their vibrissae were stimulated and, in response, they rear and turn to either side (left or right). Each session lasted 10 min, and the turns in each direction were recorded. The LI was calculated with the following formula, as described previously by Balkaya and Endres, 2010^[^ ^92^ ^]^

(1) $$\mathrm{LI}\;=\;\frac{\mathrm{TL}-\mathrm{TD}}{\mathrm{TD}+\mathrm{TL}}$$

where TL means turn left (stroke‐affected side), and TD means turn right (nonaffected side).

### MCAO Mouse Model—Infarct Volume Measurement by MRI

Ischemic lesion size was quantified by MRI (Bruker 7T PharmaScan 70/16) on day 1 and day 13 after MCAO. Analysis software (AnalyzeDirect, Overland Park, USA) was used to manually define the infarct size. After focal ischemia, cerebral edema is a commonly observed pathology that must be accounted for when measuring the infarct size. Lesion volumes were determined by computer‐aided manual tracing of the lesions and corrected for the space‐occupying effect of brain edema with the following equation^[^ ^93^ ^]^

(2) $$\%\mathrm{HLVe}\;=\;\frac{2\times \mathrm{LVe}}{\mathrm{HVc}+\mathrm{HVi}}\times 100$$

where %HLVe is the edema‐corrected lesion volume as a percentage of the hemispheric volume, HVc and HVi represent the contralateral and ipsilateral hemispheric volumes, and LVe is the edema‐corrected lesion volume.

### MCAO Mouse Model—Stereological Evaluation

For the stereological analysis, a Zeiss AxioImager I (Zeiss, Göttingen, Germany) and StereoInvestigator Software 8.0 (MicroBrightField, Magdeburg, Germany) was used. To calculate the infarct volume and the number of surviving (NeuN‐positive) neurons in the infarct area, a series of brain sections of 60 µm in thickness was used: Every 360 µm was sampled, starting from the first section with an obvious infarct (as defined by the Neurotrace 435 staining) and ending with the last one with an obvious infarct. Then, the optical fractionator workflow provided by the software was used to extrapolate the sampled subvolumes and thus estimate the volume of the entire cell population. A virtual space called an optical dissector was used, and counting rules were followed to prevent overestimating. In each brain section, the infarcted area that was still present (infarct volume) and the infarcted area that was missing because of cyst formation after necrosis (ischemic core volume) were separately outlined. Then, the neuronal density within the infarcted volume and the neuronal density on the intact contralateral (control) side were estimated and the ratio between the two densities were calculated.

### MCAO Mouse Model—GFAP and Iba1 Fluorescence Signal Analysis

Three sections per brain were imaged with an Apotome.2 fluorescence microscope (Zeiss, DE), 4× objective, and mosaic function. The images were then analyzed in Image J (National Institutes of Health, Bethesda, MD). Briefly, after background removal, the ischemic area defined by a high Iba1 signal was outlined, and the mean fluorescence intensities of the Iba1 and GFAP signals in that area were measured. The rest of the hemisphere ipsilateral to ischemia (excluding the ischemic area and ventricles) was also outlined, and the mean fluorescence intensity of both markers was measured. Last, the contralateral hemisphere (ventricles excluded) was outlined, and the mean fluorescence intensity for both markers was measured.

### Endovascular Model of Ischemic Stroke in Swine

Animal procedures were approved by the local Ethics Committee of the Warsaw University of Life Sciences in Warsaw, Poland (WAW2/046/2021). Eleven male juvenile (5‐month‐old) domestic pigs (mean weight, 35 kg) were included in the study. Endovascular procedures were performed in a dedicated large animal surgical suite located in the vicinity of the MRI scanner at the School of Life Sciences, Warsaw, Poland. Animals were randomly divided into two groups: GA treatment (*n* = 6) and placebo control (*n* = 5). To minimize stress, the animals were acclimated after arrival at the animal facility for at least 1 week.

### X‐Ray Guided Endovascular Procedure

Anesthesia was induced with atropine (0.05 mg kg^−1^ i.m., Polfa, Poland), xylazine (3 mg kg^−1^ i.m., Vetoquinol, Poland), and ketamine (6 mg kg^−1^ i.m., Vetoquinol, Poland). Before intubation, animals received propofol (5 mg kg^−1^ per hour i.v., B. Braun Melsungen AG, Germany), and after intubation, anesthesia was continued with isoflurane (1% to 3%, Baxter, USA). During the entire procedure, vital parameters were monitored (blood pressure, respiratory rate, and heart rate). Analgesia was provided every 4 h with butorphanol (0.2 mg kg^−1^ i.m., Zoetis, Poland). The endovascular procedure was performed under sterile conditions. The introducer (4F) was inserted percutaneously into the femoral artery. Then, the endovascular catheter (4F, 110 cm, Vertebral, Balton) was advanced to the right ascending pharyngeal artery (APA) over a hydrophilic guidewire (Balton) by using contrast agent (Iomeron, 400 mg J mL^−1^ Medicover) and a C‐arm. Then, the catheter was secured in place, and the animal was transferred from the surgical suite to a 3T MRI scanner (GE Healthcare).

### Magnetic Resonance Imaging, Infarct Induction, and Treatment Administration

The MRI protocol included the following: T2w (TE/TR = 98/4381) for anatomical reference, T1 and T1+contrast (TE/TR = 14/500), PWI, SWI (TE/TR = 33/42) to detect thrombus, and DWI to visualize the infarct core (with multi *b* value; TE/TR = 99/3979). Dynamic gradient‐recalled echo sequences/echo planar imaging (GRE/EPI, TE/TR = 52/3200) were also used for real‐time MRI assessment of intravenous perfusion and trans‐catheter cerebral perfusion. After acquiring the baseline scans, intravenous sodium nitroprusside (5 mg mL^−1^, Sigma‐Aldrich) was administered to reduce blood pressure, with an initial bolus of 0.5 mL and continuous infusion at 10 mL per hour for 30 min. Thrombin (200 U mL^−1^, Biomed, Poland) was mixed with Gadovist at a 1:20 volume ratio, and then a sodium nitroprusside 500 *μ*L bolus of a mixture was immediately injected intra‐arterially (400 mL per hour) with an infusion pump. After thrombin administration, SWI and DWI scans were performed to confirm blockage. Two hours after thrombin injection, tPA (20 mg at a concentration of 1 mg mL^−1^ with an infusion speed of 400 mL per hour) was injected intraarterially. Five minutes after tPA injection, the experimental and vehicle groups received GA or vehicle, respectively, intra‐arterially. Then, the animals were removed from the scanner, and the catheter and introducer were removed. After recovery from anesthesia, animals were returned to the livestock housing, except for 2 animals that had to be transferred to the intensive care unit. The second dose of treatment (GA vs vehicle) was administered intravenously after 24 h. Follow‐up MRI scans were performed 7 and 28 days after stroke induction.

### Blood Sample Tests

Before surgery and during MRI follow‐ups, blood samples were collected for morphology and gasometry. Morphology included white blood cell count (WBC), red blood cells (RBCs) and platelets (PLT), hemoglobin level (HGB), hematocrit (HCT), mean red blood cell volume (MCV), mean corpuscular hemoglobin (MCH), mean hemoglobin concentration (MCHC), red blood cell content, red blood cell distribution width (RDW), mean platelet volume (MPV), platelet distribution width (PDW), and plateletcrit (PCT). Blood gas analysis was aimed at assessing the acid–base balance. Blood pH, partial pressure of carbon dioxide (pCO2), partial pressure of oxygen (pO2), the concentration of bicarbonate (cHCO3), oxygen saturation (SO2), level of Na+, K+, Ca++, Cl−, total carbon dioxide concentration (cTCO2), anion gap (Agap and Agapk), hematocrit (Hct), hemoglobin (cHgb), glucose (Glu), lactate (Lac), and creatinine (Crea) were measured.

### Postoperative Care

For 24 h after the procedure, animals were kept in solitary confinement to allow them to recover and enable to assess their condition before they were returned to the herd. Animals received antibiotic prophylaxis (Penicillin LA 24 000 U kg^−1^) and analgesic therapy (butorphanol 0.4 mg kg^−1^ and metamizole 30 mg kg^−1^) administered intramuscularly. After the operation, the animals were in good general condition with easily detectable deficits. Two pigs (one from the control group and one from the GA group) required intensive care for 3 days after the procedure because of contralateral paralysis. In addition, one pig in the control group died 24 h after stroke induction.

### Behavioral Assessment

Animals were subjected to neurological assessment with the Neurological Examination Grading Scale, as previously described.^[^ ^38^ ^]^ Testing was done on day 0 (before the procedure) and day 1, day 2, day 3, day 7, day 14, day 21, and day 28.

### Euthanasia

After the last MRI follow‐up scan at 28 days, animals received a lethal dose of sodium pentobarbital (Euthasol, Fatro, Poland). After obtaining access to the heart, transcardial perfusion was performed. Perfusion pressure was maintained at 120–140 mm Hg and included a prewash with 5% sucrose and 4% PFA solution. The brains were extracted from the skull and placed in the PFA solution for postfixation.

### Statistical Analysis

All data were analyzed with GraphPad Prism 6 (GraphPad Software, San Diego, USA). The statistical tests used for each analysis are explained in the figure legends. Generally, *p* values of less than 0.05 were considered statistically significant. Data were presented as mean ± SEM. A total of 3 biological replicates (*n* = 3) consisting of 5 technical replicates each (mean of 5 wells per condition per plate) were used in the in vitro OGD model for evaluation of necrosis (ratio of PI+/NeuN+ neurons, 30 min after OGD; Figure 1C; one‐way ANOVA followed by Tukey's multiple comparisons test) and apoptosis (number of NeuN+ neurons normalized to normoxia, 72 h after OGD; Figure 1D; unpaired *t*‐test); a total of *n* = 6 was used for the calcium influx experiments (ΔFluo‐4‐ fluorescence normalized to timepoint 0; Figure 1E; two‐way ANOVA followed by Sidak's multiple comparisons test); and a total of *n* = 12 was used for the glutamate‐dependent calcium influx experiments (ΔFluo‐4‐fluorescence normalized to the timepoint pretreatment; Figure 1F; one‐way ANOVA followed by Tukey's multiple comparisons test).

In the in vivo GCI model, the group sizes were as follows: *n*_sham_ = 16, *n*_vehicle_ = 9, and *n*_GA_ = 7. The number of neurons stained with cresyl violet is presented (Figure 2C). One‐way ANOVA followed by Dunnett's multiple comparisons test was performed.

In the in vivo MCAO model, the group sizes and statistical tests used were described for each graph. Survival graph: Figure 3B; *n*_GA_ = 18, *n*_vehicle_ = 16, and *n*_sham_ = 6; Mantel–Cox survival test, Chi square evaluation. Infarct size day 13 versus day 1 for GA group: Figure 3D, values represent the size of infarct; *n*_GA_ = 17; paired *t*‐test. Infarct size day 13 versus day 1 for vehicle group: Figure 3E, values represent the size of infarct; *n*_vehicle_ = 10; paired *t*‐test. Stereological quantification: Figure 3F, values represent the volume in mm^3^; *n*_GA_ = 17, *n*_vehicle_ 10, and *n*_sham_ = 5; unpaired *t*‐test. Corner test: Figure 3G, values represent the laterality index; *n*_GA_ = 17, *n*_vehicle_ = 13, and *n*_sham_ = 6; one‐way ANOVA followed by Bonferroni's multiple comparisons test. Correlation between LI and infarct size: Figure 3H, *n*_GA_ = 17, *n*_veh_ = 13, and *n*_sham_ = 6; Pearson's *r* evaluation. Microglia and astrocyte activation: Figure 4, values represent the mean fluorescence intensity; *n*_sham_ = 3, *n*_veh_ = 6, and *n*_GA_ = 7; one‐way ANOVA followed by Tukey's multiple comparisons test. Endovascular model of stroke in swine: in Figure 5B, values represent probability of survival, *n*_control_ = 5 and *n*_GA_ = 6; in Figure 5C, values represent neurological score, *n*_control_ = 4 and *n*_GA_ = 6, and in Figure 5D, values represent the lesion volume in the T2 MRI sequence normalized to the lesion volume in the ADC sequence (ischemic core on day 0), *n*_control_ = 4 and *n*_GA_ = 6.

## Conflict of Interest

F.P.‐M. has a patent pending on the use of glycolic acid in ischemia. D.G., P.W., and M.J. are co‐owners of Ti‐com, which performed the swine experiments. All other authors declare no competing interests.

## Author Contributions

A.C. and D.B. contributed equally to this work. F.P.‐M. designed and coordinated the study. F.P.‐M., I.R.Á., and C.F.‐S. designed the OGD experiments. F.P.‐M. and N.P. designed the GCI experiments. A.M., K.W., and F.P.‐M. designed the MCAO experiments. M.J., P.W., Z.G., D.G., and F.P.‐M. designed the swine experiments. I.R.Á. and Y.D. performed the OGD in vitro experiments, including imaging and analysis. N.P. coordinated the GCI experiments. U.M. performed the GCI operations and assessment of neuronal survival. A.M., D.B., and K.W. coordinated the MCAO experiments. D.B., K.W., and C.D. performed the MCAO operations and GA administration. L.W., M.A., and A.C. performed the assessment of infarct size by MRI and functional tests and analyzed them for the MCAO mouse model. A.C. and Y.D. performed the histological processing and analysis of the MCAO brain tissue. A.C. performed the stereological analysis of the MCAO brains. P.W., Z.G., and D.G. performed the endovascular swine model experiments. M.J., P.W., and D.G. analyzed the data from the swine model. F.P.‐M., A.M., N.P., A.C., D.B., and U.M. wrote the manuscript. All other authors critically revised and corrected the manuscript.

## Supporting information

Supporting Information

Supplemental Video 1

## Data Availability

The data that support the findings of this study are available from the corresponding author upon reasonable request.
