# Supplementary material for: A Primeval Mechanism of Tolerance to Desiccation Based on Glycolic Acid Saves Neurons in Mammals from Ischemia by Reducing Intracellular Calcium‐Mediated Excitotoxicity
Source: Adv Sci (Weinh). 2021 Dec 14;9(4):2103265. doi: 10.1002/advs.202103265 (PMC8811841; doi:10.1002/advs.202103265)
Supplement: Supplementary file 1 — Supporting Information [file ADVS-9-2103265-s002.pdf]

ADVANCED SCIENCE
Open Access

Supporting Information

for *Adv. Sci.,* DOI: 10.1002/advs.202103265

A PRIMEVAL MECHANISM OF TOLERANCE TO DESICCATION BASED ON GLYCOLIC ACID SAVES NEURONS FROM ISCHEMIA IN MAMMALS BY REDUCING INTRACELLULAR CALCIUM-MEDIATED EXCITOTOXICITY

Alexandra Chovsepian*[1] Daniel Berchtold*[2], Katarzyna Winek[2, ¶], Uta Mamrak[3], Inés Ramirez Álvarez[4,5,‡], Yanina Dening[1], Dominika Golubczyk[6], Luis Weitbrecht[2], Claudia Dames[2], Marine Aillery[1,+], Celia Fernandez-Sanz[4,5,†], Zdzislaw Gajewski[7], Marianne Dieterich[4,5], Miroslaw Janowski[8], Peter Falkai[1], Piotr Walczak[8], Nikolaus Plesnila[3,5], Andreas Meisel[2], Francisco Pan-Montojo[1,4,5,ψ]

A PRIMEVAL MECHANISM OF TOLERANCE TO DESICCATION BASED ON GLYCOLIC ACID SAVES NEURONS FROM ISCHEMIA IN MAMMALS BY REDUCING INTRACELLULAR CALCIUM-MEDIATED EXCITOTOXICITY

Alexandra Chovsepian*[1] Daniel Berchtold*[2], Katarzyna Winek[2, ¶], Uta Mamrak[3], Inés Ramirez Álvarez[4,5,‡], Yanina Dening[1], Dominika Golubczyk[6], Luis Weitbrecht[2], Claudia Dames[2], Marine Aillery[1,+], Celia Fernandez-Sanz[4,5,†], Zdzislaw Gajewski[7], Marianne Dieterich[4,5], Miroslaw Janowski[8], Peter Falkai[1], Piotr Walczak[8], Nikolaus Plesnila[3,5], Andreas Meisel[2], Francisco Pan-Montojo[1,4,5,ψ]

SUPPLEMENTAL DATA

SUPPLEMENTARY FIGURE LEGENDS

Supplementary figure 1 Ischemia-induced mortality, body weight ecolution, body surface temperature and health scores were not affected by GA treatment. A) Percentage of surviving mice: By day 5 post-operation 76,4 of vehicle-treated mice survived while 94,44% of GA-treated mice survived, however the difference between groups was not statistically significant (unpaired t-test between vehicle and GA, p=0.136; Mantel-Cox survival test: Chi square= 3.411, p=0.181 nGA=18, nveh=17, nsham=6) B) Weight measurements: By day 1 post-ischemia/sham operation mice lost 10-15% of their initial weight. Sham mice started gaining weight directly afterwards and returned to their initial weight by day 6. MCAo mice in both GA or vehicle groups continued losing weight until day 4 and never returned to their preoperative weight. The differences between GA and vehicle group were not significant (2-way ANOVA follwed by Tukey’s multiple comparisons test, p >0,05) C) Health score: Each day, 6 different parameters

(behaviour, posture, fur, eyes, boody temperature and body weight) were assessed for all groups. A score between 0 (good clinical state) and 2 (bad clinical state) in each category was assigned to each mouse. No significant differences observed between GA and vehicle groups, despite the GA group scores were closer to the ones of the sham group (2-way ANOVA, followed by Tukey's multiple comparisons test, p >0,05). D) Body temperature: The body temperature of mice remained stable through the entire experiment in all groups, ranging between 33°C and 35°C. Data displayed as mean±SEM (2-way ANOVA, p>0.05). n_{GA}=17, n_{veh}=13, n_{sham}=6

Supplementary figure 2 Size of infarct measured by MRI on day 1 or day 13 after surgery. No significant differences between GA and vehicle-treated group on day 1 or day 13 (1-way ANOVA followed by Tukey's multiple comparisons test; day1: p>0.99, day13: p>0.99). Data shown as individual measurements, lines indicate the mean and error bars represent the ±SD.

Supplementary figure 3 No significant changes in motor function were detected by the pole test after MCAo. A) Time until mice turn completely downward did not differ between sham, ischemia+vehicle and ischemia+ GA groups. B) Time until mice descend the pole and touch the floor tended to be lower in the sham group but the difference was not significant. Statistical evaluation: 1-way ANOVA, followed by Tukey's multiple comparison's test; Data displayed as mean±SEM; n_{GA}=17, n_{veh}=13, n_{sham}=6.

Supplementary figure 4 Gait analysis using the Catwalk test revealed no significant differences in the tested parameters between baseline, GA and vehicle treatment. A) No significant difference in run duration between groups (p=0,91). B) The base of support (distance between right and left forelimb) did not differ between groups (p=0,10). C) Print position (distance between the two limbs of the same side) also did not differ between groups (0,1379). D, E) Phase dispersion measured as contact of the target paw in relation to step cycle of the anchor paw did not differ between groups. D) diagonal phase dispersion (right forelimb to left

hindlimb; p= 0,14 and left forelimb to right hindlimb; p=0,10), E) non-diagonal phase dispersion (all other comparisons; p>0.1). F) Stride length, measured as the distance between two successive steps with the same paw was not significantly altered by MCAo for neither of the treatment groups (p>1.2). All parameters were statistically evaluated using 1-way ANOVA test followed by Tukey's multiple comparisons test; Data displayed as mean, error bars: minimum and maximum values; $n_{baseline}$= 36 n_{GA}=17, n_{veh}=13,n_{sham}=6.

Supplementary figure 5. Example images of brains sections 14 days after MCAo, showing the infarct areas. First (A) and last (B) brain section used for stereological quantification from a MCAo-operated mouse, treated with vehicle. First (C) and last (D) brain section used for stereological quantification from a MCAo-operated mouse, treated with GA. Red channel: NeuN staining, Green channel: Neurotrace staining; Red dashed lines: infarct area, Yellow dashed lines: missing infarct area.

Supplementary figure 6. A) Left: Example confocal image of an MCAo brain section with Neurotrace (green) staining, showing the missing (partially detached, red outlined) tissue and the non-detached ischemic, blue outlined area. Right: T2-weighted MRI image on day 13 post-MCAo, showing the same brain at approximately the same level. The areas corresponding to the ones in the confocal image are again outlined in red and blue, while a similar contralateral area outlined in green is used as non-ischemic control. B) Graphic showing the correlation between the infarct volume measured by MRI on day 13 post-MCAo (corresponding to the sum of the red and blue outlined areas shown in A, right) (x axis) and the ischemic core volume measured by stereology (corresponding to the red outlined area shown in A, left) (y axis) (Pearson's r= 0.5488; p=0.0021). C) Quantification of signal intensity in the T2-weighted MRI images (as shown in A, right). The mean T2 signal intensity was significantly higher in the red outlined area corresponding to the completely or partially detached tissue when compared to

the blue outlined non-detached ischemic and green outlined contralateral tissue (Repeated Measures 1-way ANOVA followed by Tukey's multiple comparisons test, p=0,0003; n=6).

Supplementary figure 7. GA treatment did not increase neuronal survival inside the infarct and did not affect the infarct size. A) Cell density of all brains, estimated using stereology as the number of $NeuN^+$ cells inside the infarct divided by the infarct volume (1-way ANOVA, p=0,3583; n_{GA}=17, n_{veh}=10, n_{sham}=5. B) Cell density in mice that despite undergoing MCAo did not have an observable infarct after histological processing (1-way ANOVA, p=0,1998; n_{GA}=7, n_{veh}=3, n_{sham}=5). C) Ratio of NeuN+ cell density inside the infarct versus NeuN+ cell density inside the corresponding contralateral, intact tissue (1-way ANOVA, p=0,2154; n_{GA}=17, n_{veh}=10, n_{sham}=5). D, E) No significant differences between GA and vehicle groups in the size of the infarct (p=0,6240) and total ischemic volume (p=0,6136), respectively. F, G) No significant correlation between cell density inside the infarct and the total ischemic volume in the GA (Pearson's r = -0,1761, p= 0,6266) (F) and vehicle (Pearson's r = 0,2094, p=0,6522) (G) groups (only brains with obvious infarcts used: n_{GA}=10, n_{veh}=7).

Supplementary figure 8. Second batch of MCAo experiments with earlier substance injection. A) Percentage of surviving mice: none of the differences were statistically significant (Mantel-Cox survival test: Chi square=0,7924 , p= 0,6729 n_{GA}=14, n_{veh}=14, n_{sham}=2). B) Body weight evolution: 2-way ANOVA followed by Tukey's multiple comparisons test showed significant difference only between vehicle and sham groups on day 3 post-stroke (mean diff.= -2,925, $p<0.05$). C) Health score: 2-way ANOVA showed no significant difference between groups (p= 0,6407). D) Body surface temperature: 2-way ANOVA showed no significant difference between groups (p=0,6164).

Supplementary figure 9. Second batch of MCAo experiments with earlier substance injection. A) Significantly reduced infarct size on day1 post-MCAo compared to batch 1 in the untreated group (unpaired ttest, mean diff= -9,018 ± 3,733, p= 0,0225) B) No significant difference in infarct size between MCAo (treated or non-treated) and sham groups (1-way ANOVA, p=0,1478).

Supplementary figure 10. Second batch of MCAo experiments with earlier substance injection.

A) No significant difference between groups in the corner test performance (1-way ANOVA, p= 0,0889) B) No significant difference between groups during pole test in the time to turn (1-way ANOVA, p=0,3613) or time to reach the floor (1-way ANOVA, p=0,0629).

Supplementary figure 11. Evolution of blood parameters in swine model of stroke

A) Blood parameter values on day 7 normalized to the values of day 0 (before stroke). No statistically significant differences between GA-treated and control animals were observed for any of the examined parameters (Multiple ttests; p>0.05). B) Blood parameter values on day 28 normalized to the values of day 0 (before stroke). No statistically significant differences between GA-treated and control animals were observed for any of the examined parameters (Multiple ttests; p>0.05). WBC: White blood cells; RBC: Red blood cells; HGB: Hemoglobin; HTC: hematocrit; MCV: mean red blood cell volume; MCH: mean corpuscular hemoglobin; MCHC; mean hemoglobin concentration RDW: red blood cell distribution width; PLT: Platelets; MPV: mean platelet volume; PDW: Platelet Distribution Width; PCT: Plateletcrit; pCO2: partial pressure of carbon dioxide; pO2: partial pressure of oxygen; cHCO3: concentration of Bicarbonate, SO2: Oxygen saturation; Agap, Agapk: anion gap; Glu: Glucose; Lac: Lactose; Crea: Creatinine.

Supplementary Video 1: Exemplary video of mice performing the corner test. Min. 0:00 to min. 0:35 mouse after MCAO treated with vehicle i.p. immediately after reperfusion. Min. 0:35 to min. 1:37 video of mouse after MCAO treated with GA i.p. immediately after reperfusion.

SUPPLEMENTARY FIGURES

S1

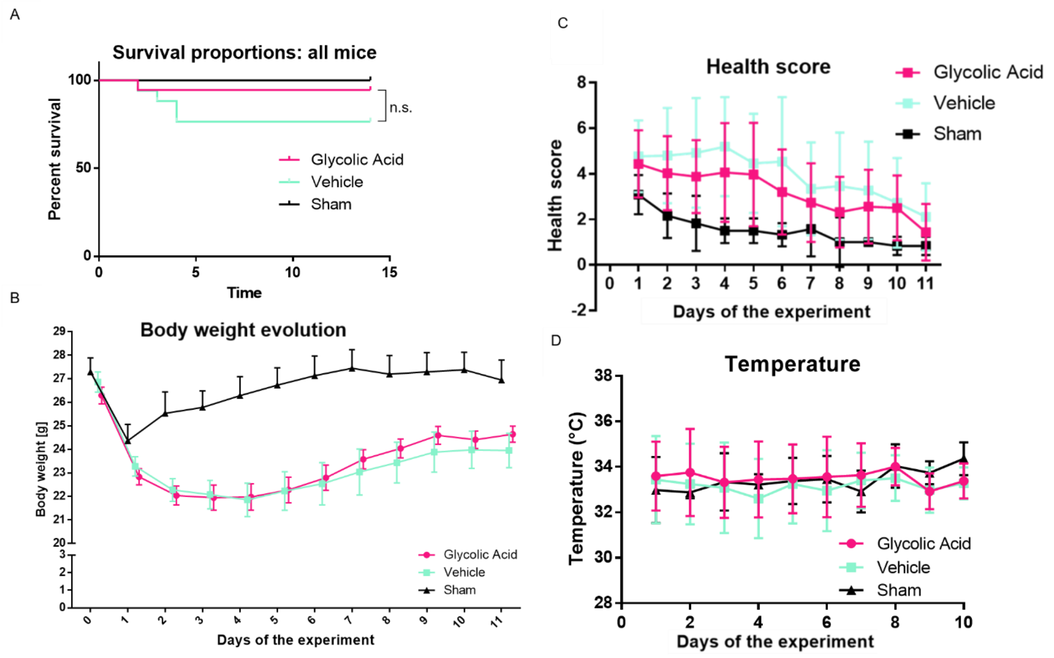

S2

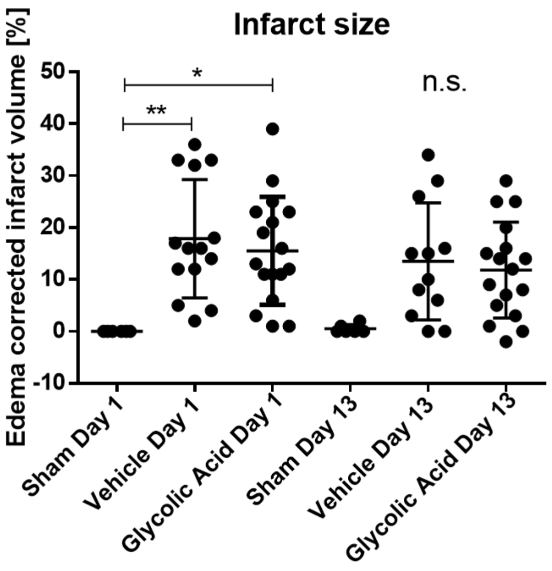

S3

Pole test

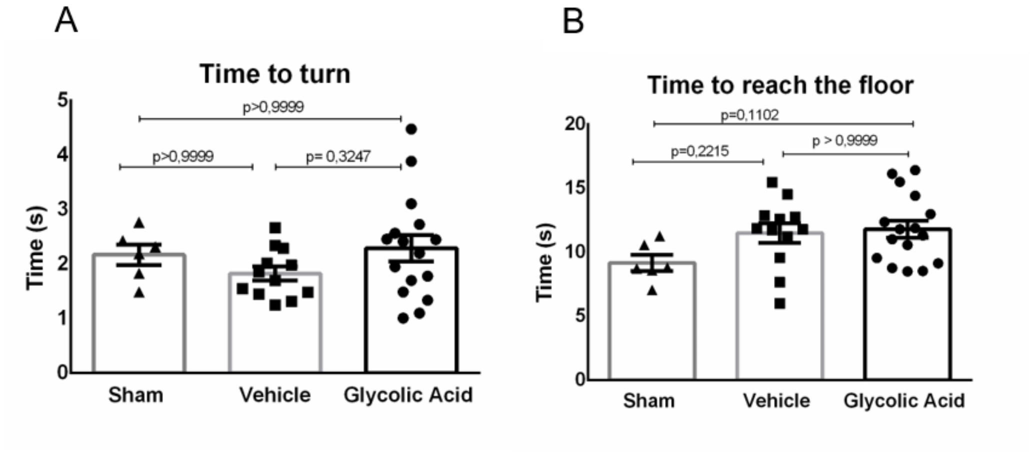

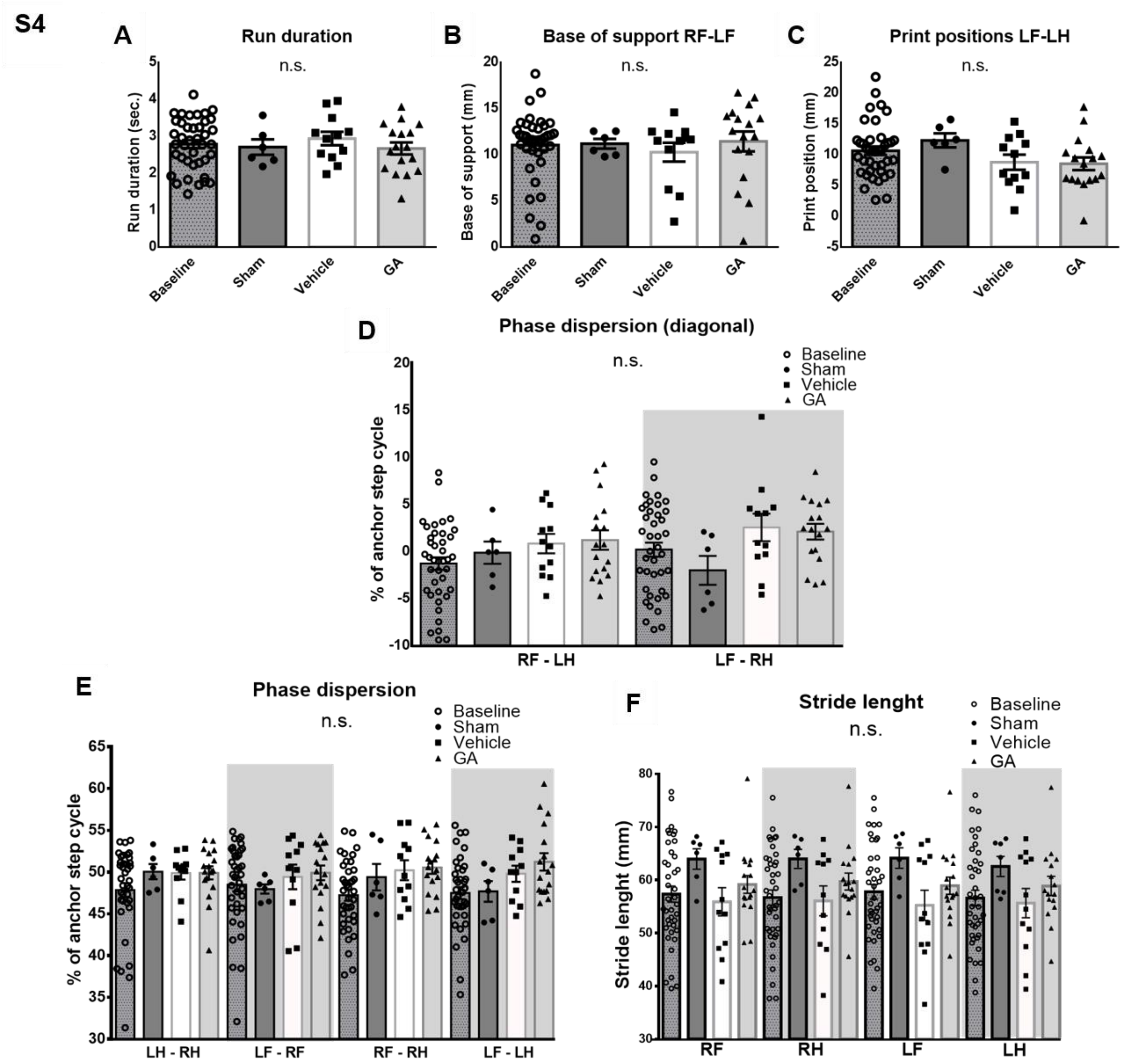
S4
A
Run duration
n.s.
Run duration (sec.)
Baseline
Sham
Vehicle
GA
B
Base of support RF-LF
n.s.
Base of support (mm)
C
Print positions LF-LH
n.s.
Print position (mm)
D
Phase dispersion (diagonal)
n.s.
% of anchor step cycle
RF - LH
LF - RH
E
Phase dispersion
n.s.
% of anchor step cycle
LH - RH
LF - RF
RF - RH
LF - LH
F
Stride lenght
n.s.
Stride lenght (mm)
RF
RH
LF
LH

**Supp. Table 1. CatWalk parameters**			Baseline	Vehicle (Mean ± SD)	GA (Mean ± SD)	Sham (Mean ± SD)
C57Bl6 mice : parameters	Definition	Paw	Day 1	Dy 10	Day 10	Day 10
Spatial characteritics						
Maximal contact area (mm²)	Area of a paw print at maximal walking surface contact	RF	22,54 ± 6,48	19,69 ± 7,06	19,57 ± 5,32	16,7 ± 4,37
		RH	22,77 ± 5,94	17,15 ± 7,17*	17,46 ± 5,55*	14,96 ± 2,69*
		LF	23,33 ± 6,11	23,52 ± 5,61	23,21 ± 6,82	19,02 ± 4,97
		LH	22,45 ± 6,94	24,21 ± 8,85	20,58 ± 6,68	17,08 ± 2,14
Kinetic characteritics						
Run duration (s)	Time for passing the walkway		2,79 ± 0,69	2,93 ± 0,63	2,67 ± 0,66	2,71 ± 0,51
Normalized swing speed (mm)	Swing speed x run duration	RF	1320,66 ± 213,71	1199,77 ± 232,95	1216,76 ± 122,65	1291,43 ± 176,79
		RH	1264,72 ± 317,92	1149,8 ± 196,72	1090,07 ± 121,32	1092,99 ± 102,25
		LF	1320,51 ± 291,33	1263,49 ± 112,90	1277,45 ± 159,16	1234,66 ± 130,14
		LH	1254,74 ± 306,99	1241,95 ± 244,75	1143,61 ± 108,11	1088,85 ± 55,46
Stand (s)	Duration of a paw contact with walking surface	RF	0,15 ± 0,03	0,16 ± 0,04	0,16 ± 0,04	0,18 ± 0,03
		RH	0,15 ± 0,03	0,15 ± 0,06	0,15 ± 0,04	0,16 ± 0,04
		LF	0,15 ± 0,03	0,16 ± 0,02	0,17 ± 0,04	0,17 ± 0,03
		LH	0,15 ± 0,03	0,16 ± 0,05	0,15 ± 0,03	0,16 ± 0,02
Comparative statistics						
Regularity index (%)	Regularity of gait		93,24 ± 7,58	88,68 ± 25,84	96,23 ± 3,51	97,27 ± 4,49
Base of support (mm)	Distance between paws	RF-LF	11,04 ± 3,60	10,28 ± 3,51	11,44 ± 4,41	11,18 ± 1,27
		RH-LH	22,79 ± 6,16	18,2 ± 7,05	21,19 ± 7,98	25,35 ± 1,82
Print positions (mm)	Distance of a hindpaw	RF-RH	10,46 ± 4,04	10,03 ± 4,25	9,09 ± 3,89	11,76 ± 2,92
		LF-LH	10,65 ± 4,42	8,78 ± 4,28	8,53 ± 4,28	12,32 ± 2,79
Stride length (mm)	Distance between successive steps with one paw	RF	57,36 ± 9,54	55,89 ± 9,20	59,17 ± 6,96	63,96 ± 4,72
		RH	56,7 ± 9,09	56,07 ± 9,35	59,71 ± 6,54	63,99 ± 4,40
		LF	57,77 ± 8,85	55,24 ± 9,83	58,93 ± 6,71	64,15 ± 4,73
		LH	56,61 ± 9,31	55,65 ± 9,56	58,88 ± 7,21	62,57 ± 5,02

Phase dispersions (%)	Contact of a target paw in relation to step cycle of the anchor paw	RF-LH	-1,30 ± 4,24	0,82 ± 3,55	1,20 ± 4,24	-0,15 ± 2,89
		LF-RH	0,18 ± 4,72	2,53 ± 5,06	2,09 ± 3,47	-2,03 ± 3,77
		LH-RH	46,91 ± 6,49	47,81 ± 7,58	49,9 ± 3,3	50,05 ± 2,23
		LF-RF	48,39 ± 5,08	49,73 ± 5,04	49,91 ± 3,64	47,97 ± 1,35
		RF-RH	46,75 ± 4,71	50,22 ± 3,96	50,55 ± 3,13	49,4 ± 3,9
		LF-LH	47,62 ± 4,32	49,81 ± 3,21	51,22 ± 4,31	47,69 ± 3,01
comparison between baselines and paired measurements at day 10 after MCAo (*p<0.05).						

S5

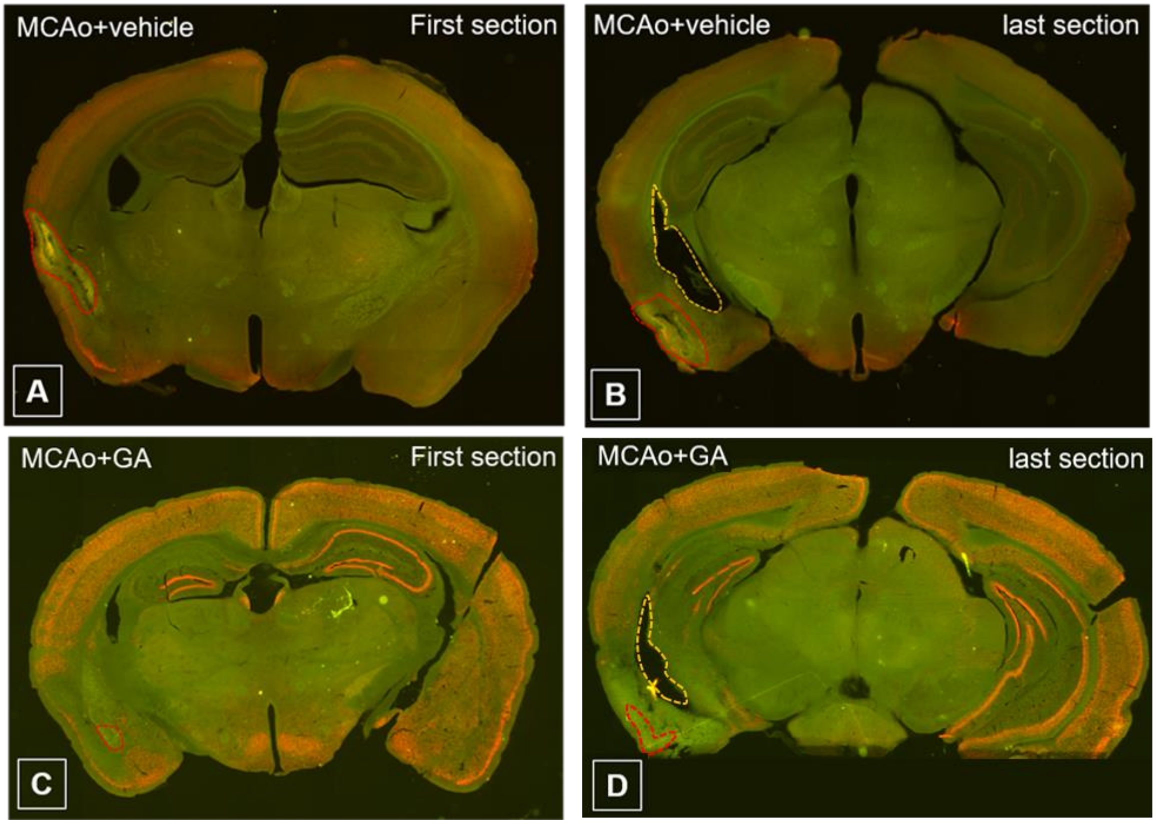

S6

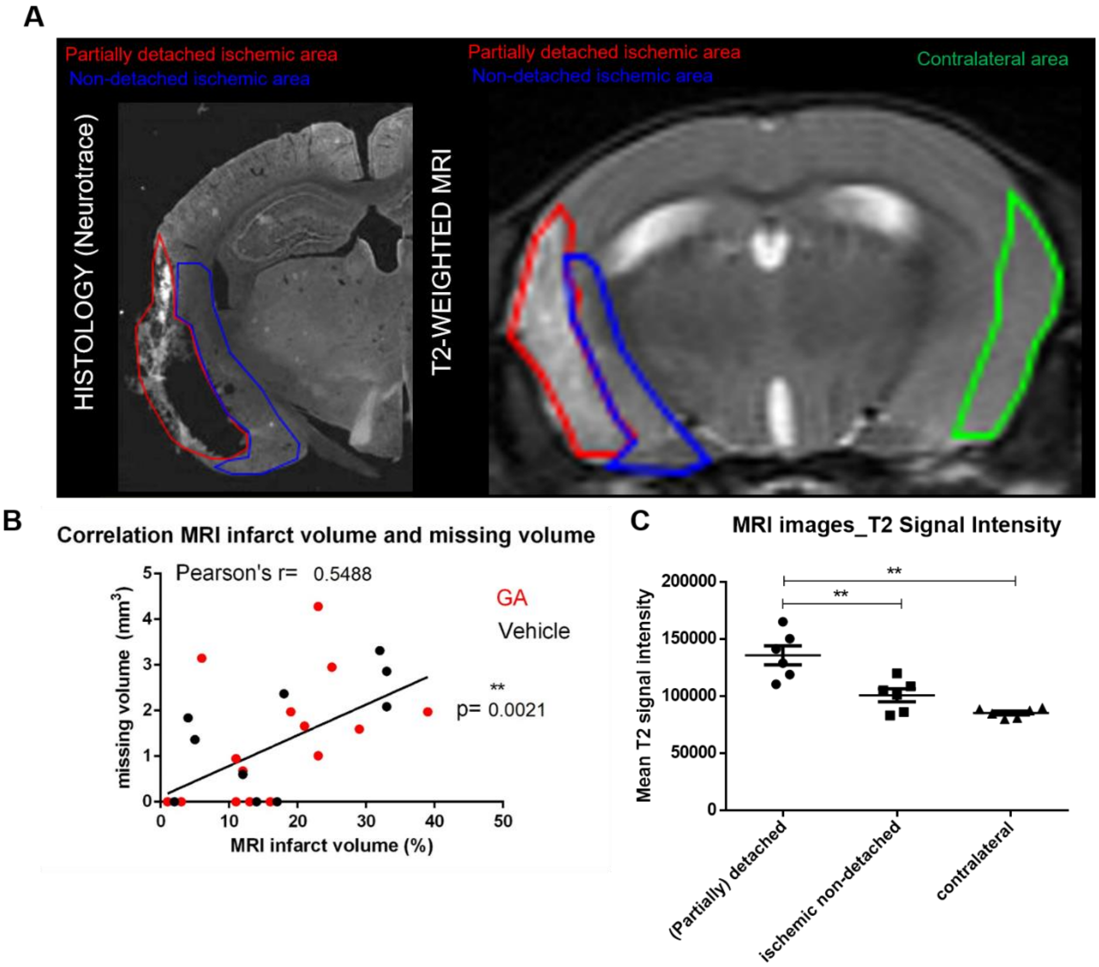
A
Partially detached ischemic area
Non-detached ischemic area
HISTOLOGY (Neurotrace)
T2-WEIGHTED MRI
Partially detached ischemic area
Non-detached ischemic area
Contralateral area
B
Correlation MRI infarct volume and missing volume
Pearson's r= 0.5488
GA
Vehicle
**
p= 0.0021
missing volume (mm3)
MRI infarct volume (%)
C
MRI images_T2 Signal Intensity
Mean T2 signal intensity
(Partially) detached
ischemic non-detached
contralateral

S7

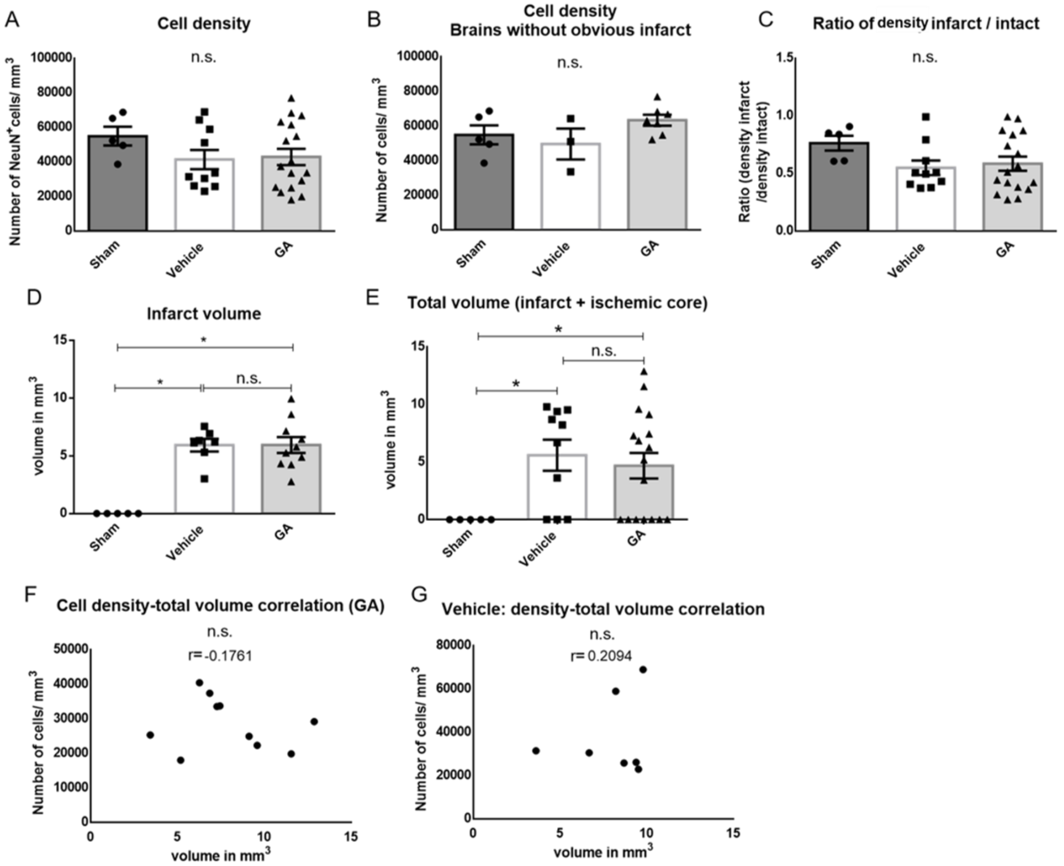
A
Cell density
n.s.
Number of NeuN+cells/ mm3
Sham
Vehicle
GA
B
Cell density
Brains without obvious infarct
n.s.
Number of cells/ mm3
C
Ratio of density infarct / intact
n.s.
Ratio (density infarct /density intact)
D
Infarct volume
volume in mm3
*
n.s.
E
Total volume (infarct + ischemic core)
F
Cell density-total volume correlation (GA)
r= -0.1761
volume in mm3
G
Vehicle: density-total volume correlation
r= 0.2094

S8

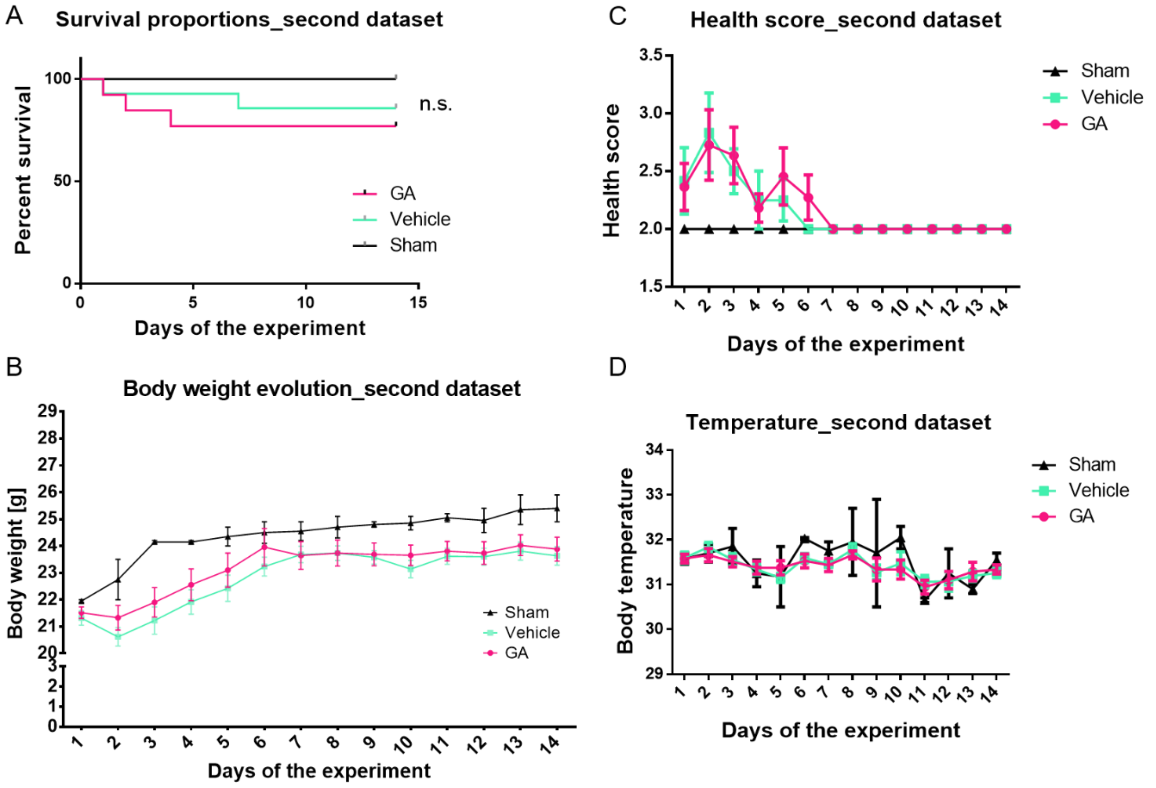
A
Survival proportions_second dataset
Percent survival
Days of the experiment
n.s.
GA
Vehicle
Sham
C
Health score_second dataset
Health score
Days of the experiment
Sham
Vehicle
GA
B
Body weight evolution_second dataset
Body weight [g]
Days of the experiment
Sham
Vehicle
GA
D
Temperature_second dataset
Body temperature
Days of the experiment
Sham
Vehicle
GA

S9

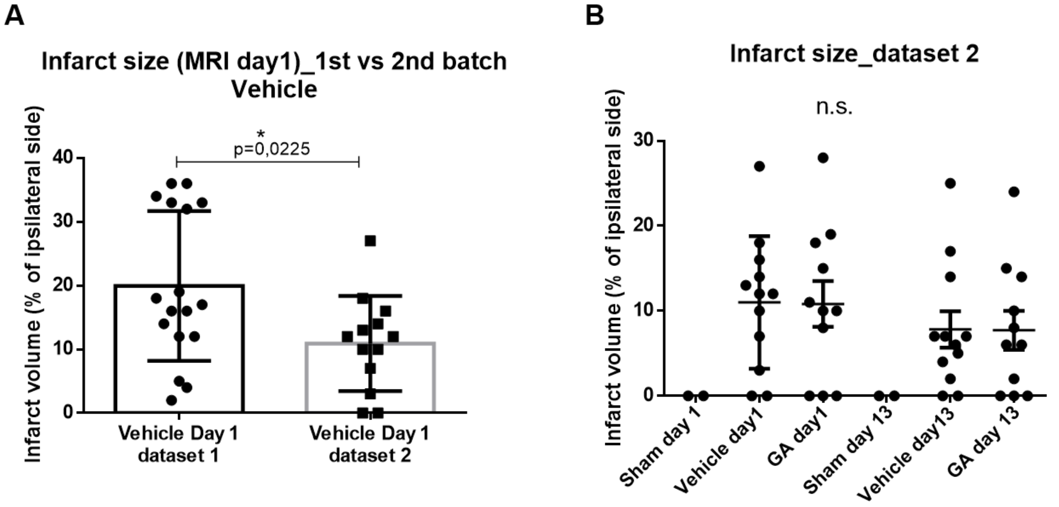
A
Infarct size (MRI day1)_1st vs 2nd batch
Vehicle
Infarct volume (% of ipsilateral side)
*
p=0,0225
Vehicle Day 1
dataset 1
Vehicle Day 1
dataset 2
B
Infarct size_dataset 2
n.s.
Infarct volume (% of ipsilateral side)
Sham day 1
Vehicle day1
GA day1
Sham day 13
Vehicle day13
GA day 13

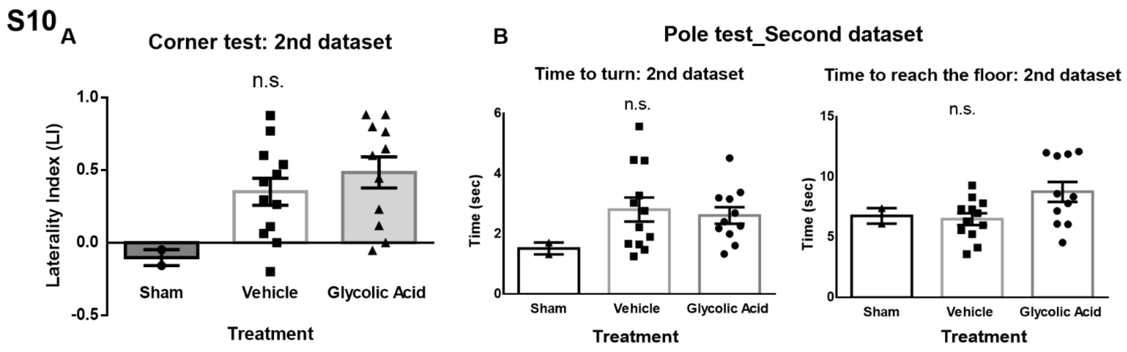
S10
A
Corner test: 2nd dataset
n.s.
1.0
0.5
0.0
-0.5
Laterality Index (LI)
Sham
Vehicle
Glycolic Acid
Treatment
B
Pole test_Second dataset
Time to turn: 2nd dataset
n.s.
6
4
2
0
Time (sec)
Sham
Vehicle
Glycolic Acid
Treatment
Time to reach the floor: 2nd dataset
n.s.
15
10
5
0
Time (sec)
Sham
Vehicle
Glycolic Acid
Treatment

S11

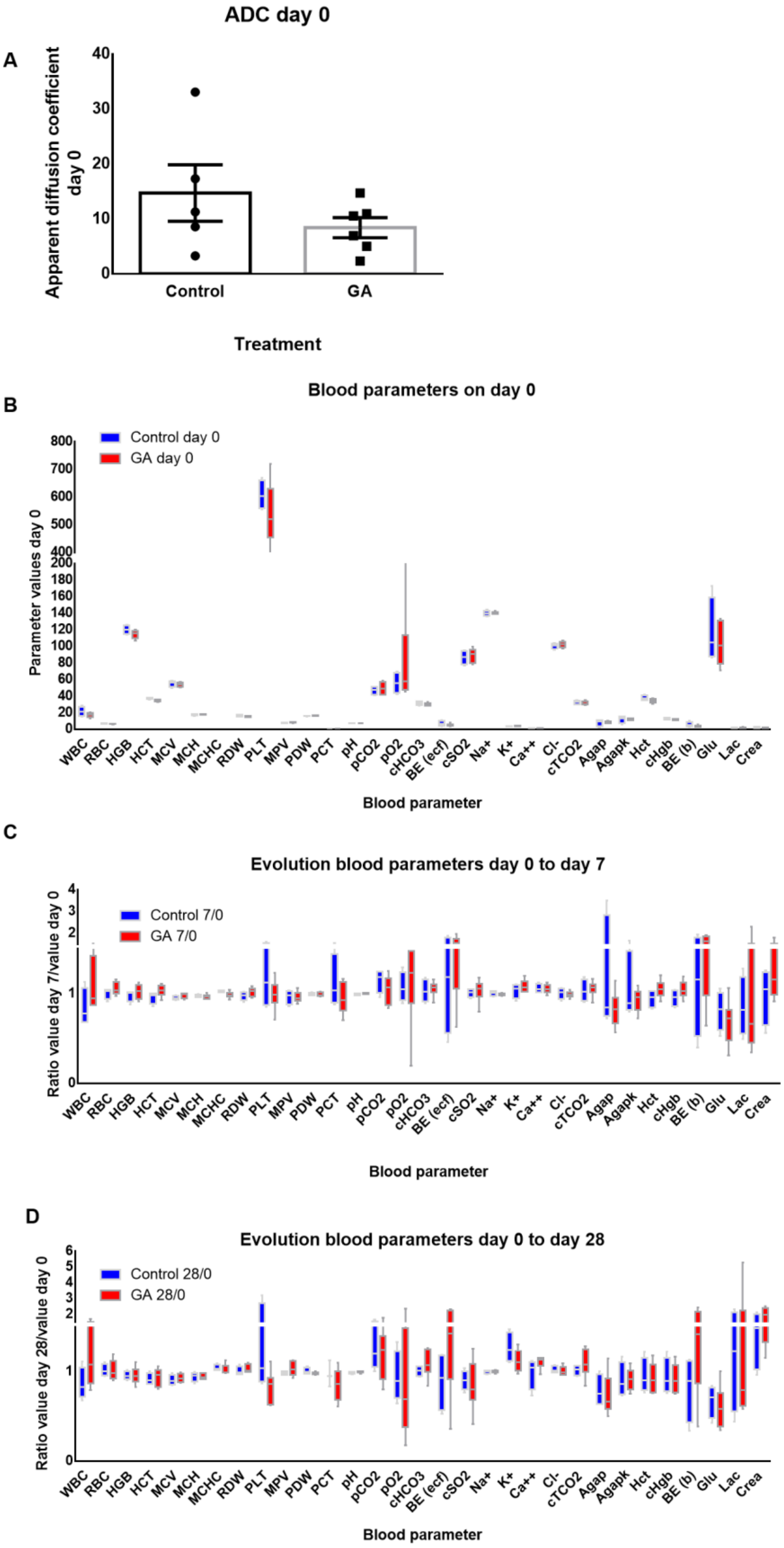
ADC day 0
A
Apparent diffusion coefficient day 0
40
30
20
10
0
Control
GA
Treatment
Blood parameters on day 0
B
Control day 0
GA day 0
Parameter values day 0
800
700
600
500
400
200
180
160
140
120
100
80
60
40
20
0
WBC RBC HGB HCT MCV MCH MCHC RDW PLT MPV PDW PCT pH pCO2 pO2 cHCO3 BE (ecf) cSO2 Na+ K+ Ca++ Cl- cTCO2 Agap Agapk Hct cHgb BE (b) Glu Lac Crea
Blood parameter
C
Evolution blood parameters day 0 to day 7
Control 7/0
GA 7/0
Ratio value day 7/value day 0
4
3
2
1
0
WBC RBC HGB HCT MCV MCH MCHC RDW PLT MPV PDW PCT pH pCO2 pO2 cHCO3 BE (ecf) cSO2 Na+ K+ Ca++ Cl- cTCO2 Agap Agapk Hct cHgb BE (b) Glu Lac Crea
Blood parameter
D
Evolution blood parameters day 0 to day 28
Control 28/0
GA 28/0
Ratio value day 28/value day 0
6
5
4
3
2
1
0
WBC RBC HGB HCT MCV MCH MCHC RDW PLT MPV PDW PCT pH pCO2 pO2 cHCO3 BE (ecf) cSO2 Na+ K+ Ca++ Cl- cTCO2 Agap Agapk Hct cHgb BE (b) Glu Lac Crea
Blood parameter

REFERENCES
